# Foot Progression Angle Modulates Three‐Dimensional Lower‐Limb Biomechanics in Flexible Flatfoot: Kinematic–Kinetic Patterns and Clinical Implications

**DOI:** 10.1002/jfa2.70126

**Published:** 2026-01-31

**Authors:** Linxiao Shen, Dong Sun, Yufei Fang, Zhenghui Lu, Xin Li, Yufan Xu, Yang Song, Chengyuan Zhu, Xuanzhen Cen, Gusztáv Fekete, Monèm Jemni, Yaodong Gu

**Affiliations:** ^1^ Faculty of Sports Science Ningbo University Ningbo China; ^2^ Ningbo No. 2 Hospital Zhejiang Engineering Research Center for New Technologies and Applications of Helium‐Free Magnetic Resonance Imaging Ningbo China; ^3^ Department of Rehabilitation Medicine Ningbo No. 2 Hospital Ningbo China; ^4^ Faculty of Engineering University of Pannonia Veszprém Hungary; ^5^ Department of Materials Science and Mechanical Engineering AUDI Hungaria Faculty of Engineering Széchenyi István University Győr Hungary; ^6^ Department of Biomedical Engineering Faculty of Engineering The Hong Kong Polytechnic University Hong Kong China; ^7^ Centre for Mental Health Research in Association with the University of Cambridge Cambridge UK; ^8^ The Carrick Institute Cape Canaveral Florida USA; ^9^ Research Institute of Sport Science Hungarian University of Sports Science Budapest Hungary

**Keywords:** biomechanics, flexible flatfoot, foot progression angle, machine learning

## Abstract

**Introduction:**

Foot progression angle affects gait and lowerlimb alignment. Altered angles may increase knee and ankle loading and produce tissue loading patterns previously linked to musculoskeletal injury. This study investigates how different foot progression angles modify knee and ankle biomechanics in young adults with flexible flatfoot.

**Methods:**

28 participants (aged 18–35 years) with flexible flatfoot completed gait trials under three foot progression angle conditions. Kinematic and kinetic variables were analyzed using one‐dimensional statistical parametric mapping. A 1D convolutional neural network was applied to classify progression angle patterns based on flexible flatfoot severity and gait biomechanics.

**Results:**

Decreasing foot progression angle reduced the ankle eversion/inversion range and knee abduction and external rotation (*p* < 0.05). Increasing foot progression angle lowered early stance ankle plantarflexion and increased knee abduction/external rotation (*p* < 0.05). Kinetically, a smaller foot progression angle reduced peak ankle plantarflexion moment and knee extension moment but increased the first peak of the knee adduction moment and rotational moment fluctuations (*p* < 0.05). A larger foot progression angle reduced rotational fluctuations and terminal stance knee extension moment (*p* < 0.05). The convolutional neural network model was most accurate for moderate flexible flatfoot cases, and ankle coronal and knee transverse biomechanics showed the strongest discriminative power.

**Conclusion:**

Modifying the foot progression angle can meaningfully alter knee and ankle loading in young adults with flexible flatfoot. Neutral or mild toe‐in angles may help mitigate excessive eversion and rotational stress, suggesting a simple noninvasive adjustment that clinicians can incorporate during gait retraining or orthotic prescription. Because biomechanical responses vary across individuals, FPA modification may be the most effective when tailored to patient‐specific gait characteristics. In addition, deep‐learning‐based gait classification shows promise for supporting personalized monitoring and guiding clinical decision‐making during rehabilitation.

## 1 Introduction

Flexible flatfoot (FFF) is a prevalent structural abnormality in foot structure, with a prevalence of approximately 23%–26.5% [1, 2, 3]. It is chiefly defined by a diminished medial longitudinal arch (MLA) and reduced arch elevation under weight‐bearing conditions [3, 4]. This structural deviation often leads to alterations in lowerlimb alignment and reduced postural stability, thereby increasing the risk of musculoskeletal injuries [5, 6, 7, 8]. Studies have shown that individuals with flat feet exhibit greater lowerlimb flexion during drop‐landing motion and have difficulty attenuating impact forces, highlighting the need for proper postural control to absorb loads and strengthen the lower extremities [9]. To better understand the biomechanical mechanisms relevant to tissue loading in flat‐footed individuals, an increasing number of studies have adopted gait analysis to examine lowerlimb loading differences under various gait patterns [10, 11, 12]. There is growing evidence linking bilateral flatfoot deformity to heightened levels of knee discomfort [13]. Gross et al. demonstrated that variations in flatfoot morphology are closely, and linearly, linked to medial tibiofemoral cartilage deterioration among elderly individuals [14], indicating a potential connection to early‐stage clinical manifestations of knee osteoarthritis (OA). Over the past few years, the foot progression angle (FPA), a key rotational parameter of gait, has gained growing attention [15, 16]. The FPA represents the angle formed between the foot's longitudinal axis and the line of progression, categorizing gait into neutral, toe‐in, or toe‐out patterns [17]. Some studies have reported that individuals with low arches tend to have increased FPA values [18]. Given that FPA reflects rotational loading characteristics of the foot [19, 20], guiding individuals with FFF to adjust their FPA may offer a novel intervention strategy for preventing knee and other lower extremity injuries.

Individuals with FFF exhibit altered lowerlimb biomechanics compared with those with neutral arches, including excessive ankle eversion and valgus‐related knee mechanics, along with changes in flexion–extension and rotational motion patterns [18, 21, 22, 23, 24]. These deviations can shift the lowerlimb mechanical axis and reshape global joint loading. FPA has been widely examined as a modifiable parameter for redistributing joint load, with toe‐in gait shown to reduce the knee adduction moment (KAM) and medial knee stress, though sometimes accompanied by compensatory changes in flexion and rotational loading [15, 16, 25]. However, current evidence mainly focuses on healthy individuals or knee OA cohorts, and the biomechanical responses of individuals with FFF under different FPA gait conditions remain unclear. Moreover, substantial interindividual variability within this population suggests that different people may respond differently to FPA modification, highlighting the need for tools capable of identifying subject‐specific gait patterns. Traditional gait‐analysis methods typically rely on discrete variables or averaged waveform parameters, which may overlook subtle spatiotemporal and multiplanar biomechanical features associated with FPA‐induced gait changes. In contrast, deep‐learning approaches—particularly convolutional neural networks (CNNs)—have shown strong capabilities in extracting high‐dimensional, joint‐coupled gait features, and classifying movement patterns without manual feature selection [26, 27, 28, 29]. Therefore, incorporating a CNN model provides an opportunity to characterize FPA‐dependent gait phenotypes more comprehensively and to support individualized gait‐modification strategies for FFF.

Individuals with FFF may be more sensitive to changes in FPA due to altered lowerlimb alignment and load distribution. These characteristics can magnify the biomechanical consequences of FPA adjustments, particularly in the coronal and transverse planes, leading to greater compensatory changes in knee and ankle joint mechanics to maintain gait stability. Therefore, this study proposes the following hypothesis: Variations in FPA will significantly affect the kinematic and kinetic parameters of the knee and ankle joints in individuals with FFF. Specifically, altered FPA patterns—including toe‐in and toe‐out orientations—are anticipated to have a more substantial impact on joint mechanics than neutral gait, reflecting a higher demand for gait compensation.

The present study explores how three representative FPAs—toe‐in (−7°), neutral (5°), and toe‐out (22°)—influence the kinematic and kinetic behavior of the ankle and knee joints in individuals with FFF [15]. The focus is on analyzing the trends in joint angles and joint moments under different FPA conditions. The outcomes of this study contribute to the evidence base needed to formulate individualized gait interventions and conservative management for people with FFF, with the goal of optimizing gait control and minimizing the risk of musculoskeletal impairments in the lower extremities.

## 2 Materials and Methods

### 2.1 Participants

**FIGURE 1:**
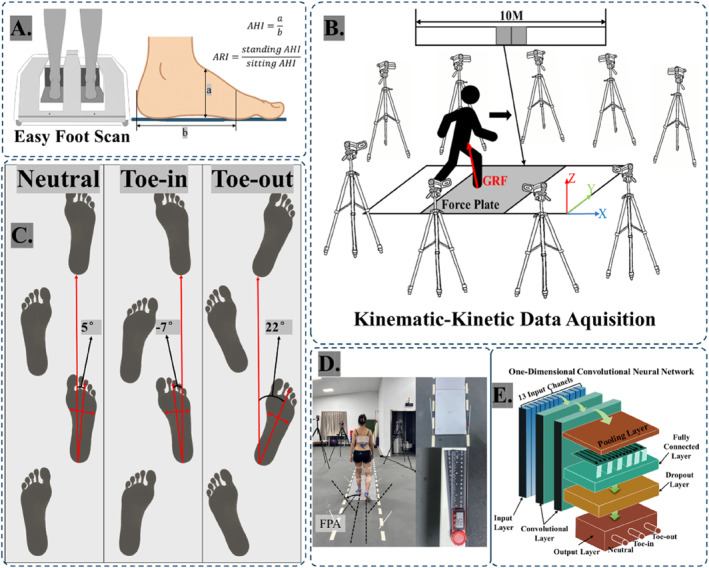
(A) Methods for foot‐type assessment and arch rigidity index calculation; (B) kinematic and kinetic data acquisition; (C) three FPAs diagram; and (D) FPA measurements were obtained via goniometry, and visual markers on the walking surface served to direct participants toward maintaining the intended gait angles. (E) Schematic diagram of one‐dimensional convolutional neural network (including 13 channels with data on flatfoot severity, angles, and torques of three planes of the ankle joint and knee joint).

G*Power software (v3.1.9.7, Heinrich Heine University, Düsseldorf, Germany) was used to carry out an a priori power analysis, aimed at identifying the minimum sample size for key statistical evaluations. The sample size estimation, based on a repeated‐measures design with ANOVA parameters (effect size *f* = 0.25, significance level *α* = 0.05, and statistical power = 0.80), suggested a minimum of 28 participants. Additionally, a CNN model was employed as part of an exploratory analysis to examine potential classification patterns. As this machine learning component was not hypothesis‐driven, it was not included in the a priori power computation. Accordingly, 28 participants with FFF (16 males and 12 females), aged 18–35 years, and had no lowerlimb injuries or disorders in the past 6 months, were recruited. Three‐dimensional plantar morphology during static standing (feet shoulder‐width apart) and sitting was measured using the easy‐foot‐scan system (OrthoBaltic, Kaunas, Lithuania) operating at 1.0 mm detail resolution, with smoothing and gap‐filling values set at 30 and 100 mm, respectively (Figure 1A). All participants were right‐side dominant in terms of lowerlimb use. Although FFF can be bilateral and asymmetric, symmetry analysis of foot morphology in the present cohort showed no significant differences between the left and right feet (Table 1). To maintain consistency and avoid potential interlimb interaction effects when comparing FPA conditions, the dominant (right) limb was selected a priori for biomechanical analysis. Based on the foot scan data, subjects exhibiting an arch index (AI) of 0.26 or higher were categorized as having flat feet. The AI was calculated as the ratio of the midfoot contact area to the total plantar footprint area, excluding the toe region [30, 31]. FFF was identified based on the arch rigidity index (ARI) equals the quotient of the standing arch height index (AHI) over its seated counterpart (Figure 1A). An ARI value of approximately 1.0 indicates a rigid flatfoot, whereas values less than 1.0 are considered indicative of a FFF [32]. AHI was calculated as the ratio of the vertical dorsum height (measured from the foot's upper surface to the supporting plane) to the truncated foot length (measured from the first metatarsophalangeal joint (MTP) to the posterior aspect of the heel) (Figure 1C) [33, 34]. Demographic characteristics, including participants' age, height, and weight, are detailed in Table 1.

**TABLE 1:** Basic characteristics of the subjects.

Characteristics		Mean ± SD		
Age (year)		23.40 ± 3.46		
Height (m)		1.70 ± 0.08		
Weight (kg)		65.69 ± 14.37		

		**Left foot**	**Right foot**	***p*‐value**
Arch index		0.27 ± 0.03	0.28 ± 0.02	0.125
Standing arch height index		0.43 ± 0.04	0.43 ± 0.03	0.720
Sitting arch height index		0.49 ± 0.07	0.47 ± 0.04	0.400
Arch rigidity index		0.88 ± 0.04	0.89 ± 0.06	0.733

**Walking speed**	**Neutral**	**Toe‐in**	**Toe‐out**	***p*‐value**
KM/H	3.32 ± 0.27	2.93 ± 0.15	3.27 ± 0.25	0.740

### 2.2 Experimental Design

A total of 38 reflective markers were positioned on specific anatomical sites across the body using the Gait‐2392 musculoskeletal framework during the experimental procedure. A total of nine infrared cameras from the Vicon motion capture system (Oxford Metrics Ltd., Oxford, UK) and two Kistler 3D force plates (Kistler Group, Winterthur, Switzerland) were used to synchronously collect kinematic and kinetic data at sampling rates of 200 and 2000 Hz, respectively, under three FPA conditions: toe‐in (−7°), neutral (5°), and toe‐out (22°) (Figure 1B). The toe‐in and toe‐out angles were selected with reference to published ranges of FPA differences observed among individuals with different static foot postures, ensuring that the imposed deviations represented biomechanically meaningful adjustments while remaining safe and feasible for participants [15]. The FPA was the angle between the foot longitudinal axis (a vector from the heel marker to the midpoint of the first and fifth metatarsal heads) and the walking direction (the displacement vector between two consecutive heel strikes) (Figure 1C) [35]. FPA was measured using a goniometer and guided by visual markers placed on the floor (Figure 1D). Participants were instructed to adopt the target FPA for each condition, a tolerance of ± 3° from the target FPA was permitted [36], which is consistent with the acceptable variability reported in controlled gait experiments; trials in which the mean FPA exceeded this tolerance were repeated. Prior to data collection for each condition, participants performed a 10‐min practice session. For each condition, participants walked at their comfortable walking speed to complete 15 successful trials, with a 10‐min rest period provided between conditions. All gait trials were performed barefoot, with no arch support or orthotic devices, to avoid shoe‐related influences and preserve the native FFF status during walking. Data acquisition focused on the stance phase of the right foot. Due to practical limitations in achieving exact FPA values during overground walking, the five data sets most closely matching the target FPA angle were selected for further analysis [15, 16].

### 2.3 Data Analysis

Using Vicon Nexus 2.15 (Oxford Metrics Ltd., Oxford, UK), raw kinematic and kinetic data underwent preprocessing. Foot contact was defined at the ground reaction force (GRF) values ≥ 10 N. To reduce noise, a zero‐phase, fourth‐order Butterworth low‐pass filter was implemented, employing cut‐off frequencies of 15 Hz for motion tracking and 50 Hz for force data. Foot contact was identified when the GRF exceeded a threshold of 10 N. A fourth‐order zero‐lag Butterworth low‐pass filter was applied to the data, with cut‐off frequencies of 15 Hz for the 3D marker trajectories and 50 Hz for the GRF data, respectively [15]. Subsequently, the filtered kinematic and kinetic data were imported into Visual3D software (C‐Motion Inc., Germantown, MD, USA) to compute joint angles and joint moments. Joint angles were calculated in Visual 3D using an X–Y–Z Cardan rotation sequence (flexion/extension; abduction/adduction; internal/external rotation), in accordance with ISB recommendations. Joint moment values were normalized to body mass × height (Nm/(kg·m)) using Visual 3D's default processing pipeline. Both joint angles and moments were resampled over 101 equally spaced time points across the stance phase.

### 2.4 Statistical Analysis

Normality of the data was first assessed. One‐dimensional statistical parametric mapping (SPM1d) was then used to perform a one‐way repeated‐measures ANOVA on joint angles and joint moments across the three FPA conditions. When the main effect was significant in the F‐test, post hoc pairwise paired t‐tests were conducted. For pairwise post hoc comparisons, the significance level was adjusted using a Bonferroni correction to account for multiple testing across the three FPA conditions. Statistical significance was set at *p* < 0.05. All analyses were conducted in MATLAB R2024a (The MathWorks, Natick, MA, USA). For each significant SPM{t} time window, a window‐based standardized effect size (Cohen's d) was calculated to quantify the magnitude and direction of the between‐condition difference.

### 2.5 CNN Modeling

To further explore how these differences manifest in practical classification tasks, this study introduces a CNN model. Feature extraction from convolutionally structured data is effectively achieved using CNNs, a form of feedforward neural network [37, 38, 39]. They have been widely applied to problems involving inputs with known grid‐like topology, such as time series (1D grids) or images (2D grids), making them a commonly adopted architecture in deep learning applications [40]. One‐dimensional convolutional neural network (1D CNN) typically employs one‐dimensional convolutional kernels to process sequential data. The convolutional layers apply multiple 1D filters that slide over the input signal to extract local features. This architecture is particularly well‐suited for learning from fixed‐length segments of a dataset, where the spatial position of features is not critical. As a result, 1D CNNs are well‐suited for applications such as time series forecasting and signal recognition.

In this study, a Python‐based 1D CNN was developed to process time‐series data while preserving temporal structures and capturing time‐dependent features (Figure 1E). The model inputs included the degree of flatfoot as well as the joint angles and moments of the ankle and knee across three anatomical planes. Flatfoot severity was determined using the built‐in arch index algorithm of the foot‐scanning system and categorized into mild (0.26–0.27), moderate (0.28–0.29), and severe (≥ 0.30). For each severity level, the data were split into training (64%), validation (16%), and test (20%) sets. To prevent data leakage, dataset partitioning was performed at the participant level rather than at the trial level; therefore, all trials from the same participant were assigned exclusively to a single subset (training, validation, or test). The training and validation datasets were employed for fitting the model and tuning its hyperparameters, whereas the test dataset was reserved for evaluating generalization performance. Separate CNN models were constructed using these features to classify FPA into three categories: neutral, toe‐in, and toe‐out. The CNN architecture consisted of three key components: convolution, pooling (max), and dense connection layers [41]. A more detailed explanation of the model is provided in the Supporting Information S1.

In addition to the single train–validation–test split, a stratified 5‐fold cross‐validation procedure was employed to further assess the robustness and generalization of the CNN model. For each joint–plane–severity condition, the dataset was randomly divided into five folds while preserving the class distribution. Each fold served once as the validation set and the remaining four folds as the training set. The mean F1 scores across the five folds are reported as cross‐validation (CV), train F1 (mean), and CV validation F1 (mean), with the difference between the two reported as ΔF1 (CV mean) to quantify overfitting.

In this study, the CNN hyperparameters were determined through a small‐scale grid search, an approach widely adopted in CNN‐based gait and biomechanical modeling research [42, 43, 44, 45]. Based on commonly reported settings in CNN‐based gait analysis studies [42, 46, 47], candidate ranges were evaluated for kernel size ∈ {3, 5, 7}, dropout rate ∈ {0.2, 0.4, 0.5}, and learning rate ∈ {1 × 10^−2^, 1 × 10^−3^, 1 × 10^−4^}. Each combination was trained for up to 120 epochs with early stopping based on validation loss. The configuration that achieved the highest validation F1 score with stable convergence—kernel size = 5, dropout rate = 0.4, and learning rate = 1 × 10^−3^—was selected for all subsequent experiments. This procedure ensured that the final model retained high feature‐learning capacity while minimizing overfitting risk.

## 3 Results

Standardized effect sizes (Cohen's d) for all significant SPM post hoc comparisons, based on the corresponding significant time windows, are provided in Supporting Information S1: Table S1 to facilitate clinical interpretation of the magnitude and direction of the observed differences.

### 3.1 Joint Kinematics

Overall, changes in FPA primarily affected ankle and knee kinematics in the coronal and transverse planes, whereas sagittal‐plane joint angles were largely preserved across conditions. Toe‐in and toe‐out gaits produced distinct frontal and rotational alignment patterns relative to neutral gait.

#### 3.1.1 Ankle Joint Angles

**FIGURE 2:**
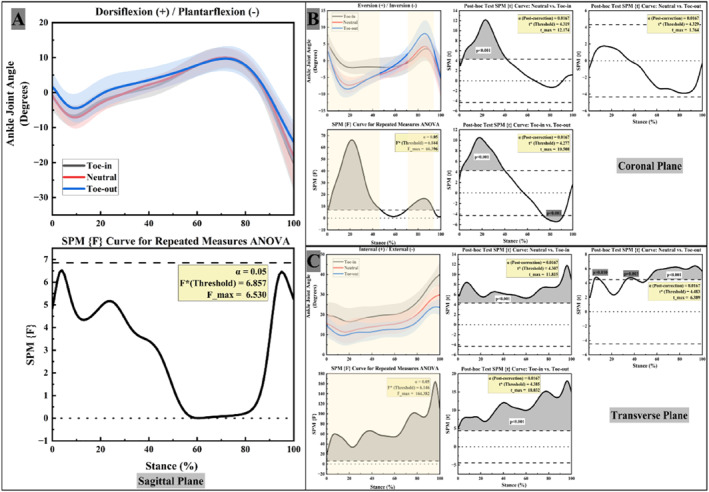
Right ankle and knee joint angles in three planes. (A) ankle joint sagittal plane angles; (B) ankle joint coronal plane angles; (C) ankle joint transverse plane angles.

Figure 2 illustrates the variations in ankle joint angles of FFF participants under different FPA conditions. In the sagittal plane (Figure 2A), ankle dorsiflexion–plantarflexion angles did not differ significantly across the three FPA conditions.

In the coronal plane (Figure 2B), significant differences among the three FPA conditions were observed during 0%–47% and 70%–94% of stance. Post hoc paired t‐tests indicated that the toe‐in gait had a significantly smaller peak inversion angle than the neutral and toe‐out conditions, whereas the toe‐out gait had a significantly greater peak eversion angle than the toe‐in condition (*p <* 0.05).

In the transverse plane (Figure 2C), the differences in ankle joint angles among the three FPA conditions were most pronounced. Throughout the stance phase, the toe‐in gait exhibited the greatest degree of ankle internal rotation, followed by the neutral gait, whereas the toe‐out gait showed the least (*p* < 0.05).

#### 3.1.2 Knee Joint Angles

**FIGURE 3:**
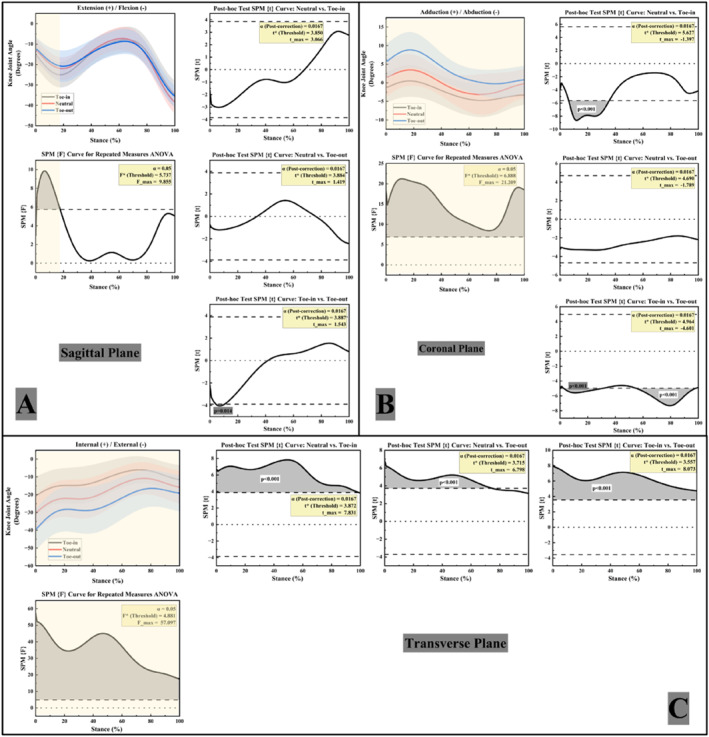
Right knee joint angles in three planes. (A) Knee joint sagittal plane angles; (B) knee joint coronal plane angles; (C) knee joint transverse plane angles.

Figure 3 illustrates the variations in knee joint angles under the three FPA conditions. In the sagittal plane (Figure 3A), knee flexion–extension angles showed a significant main effect of FPA during 0%–18% of stance. Post hoc paired t‐tests revealed that only the toe‐in gait exhibited greater knee flexion (larger knee flexion angle, KFA) than the toe‐out gait during 4%–11% of stance (*p* < 0.05).

In the coronal plane (Figure 3B), knee adduction–abduction angles showed a significant main effect of FPA across the entire stance phase. Post hoc paired t‐tests indicated that the neutral gait exhibited a significantly greater knee abduction angle than the toe‐in gait during 6%–37% of stance, and the toe‐out gait exhibited a significantly greater knee abduction angle than the toe‐in gait during 3%–28% and 56%–97% of stance (*p* < 0.05).

In the transverse plane (Figure 3C), notable differences in knee internal/external rotation angles were observed among the three FPA conditions. The toe‐out gait exhibited the greatest external rotation, followed by the neutral gait, whereas the toe‐in gait showed the least. Throughout the entire stance phase (0%–100%), the toe‐in gait showed significantly lower knee external rotation angles compared to the neutral condition (*p* < 0.05).

### 3.2 Joint Kinetics

In summary, FPA modulation had minimal impact on sagittal‐plane ankle motion but led to clear differences in coronal and transverse alignment. Toe‐in gait reduced inversion and internal rotation compared with toe‐out gait, whereas toe‐out gait was associated with greater eversion and external rotation throughout stance.

#### 3.2.1 Ankle Joint Moments

**FIGURE 4:**
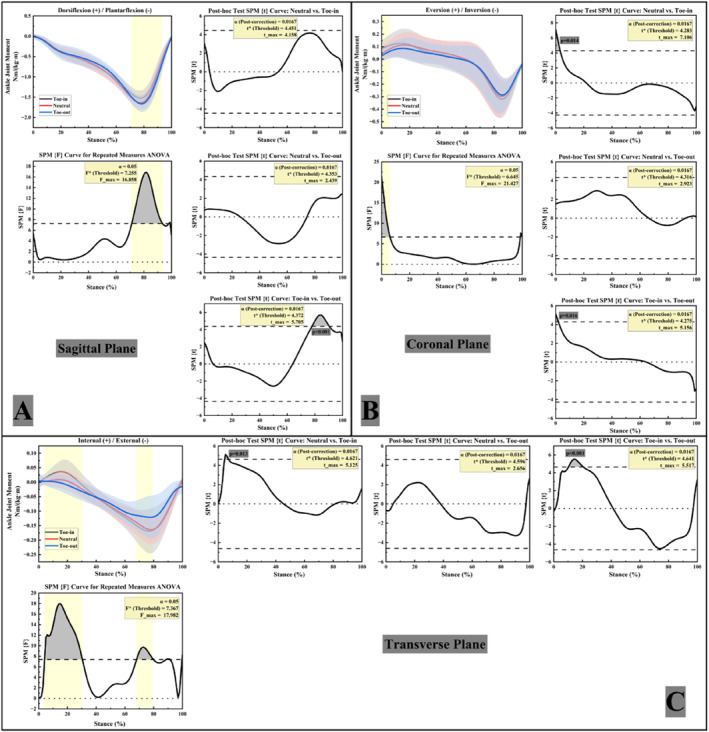
Right ankle joint moments in three planes. (A) Ankle joint sagittal plane moments; (B) ankle joint coronal plane moments; (C) ankle joint transverse plane moments.

Figure 4 illustrates the variations in ankle joint moments of FFF participants under the three gait conditions. In the sagittal plane (Figure 4A), ankle plantarflexion moments showed a significant main effect of FPA during 72%–93% of stance. Post hoc paired t‐tests indicated that the toe‐out gait exhibited significantly greater plantarflexion moments than the toe‐in gait during 78%–90% of stance (*p* < 0.05).

In the coronal plane (Figure 4B), ankle inversion–eversion moments showed a significant main effect of FPA during 0%–6% of stance. Post hoc paired t‐tests indicated that the toe‐in gait exhibited significantly greater eversion moments than the neutral gait during 0%–3% of stance and greater than the toe‐out gait during 0%–2% (*p* < 0.05).

In the transverse plane (Figure 4C), differences in ankle internal/external rotation moments were primarily observed around the moment peak intervals. The toe‐in gait exhibited the widest range of rotational moments (−0.17 Nm/kg to 0.04 Nm/kg). Ankle internal–external rotation moments showed a significant main effect of FPA during 4%–31% and 69%–80% of stance. Post hoc paired t‐tests indicated that the toe‐in gait exhibited significantly greater internal‐rotation moments than the neutral gait during 6%–7% of stance and greater than the toe‐out gait during 10%–20% (*p* < 0.05).

#### 3.2.2 Knee Joint Moments

**FIGURE 5:**
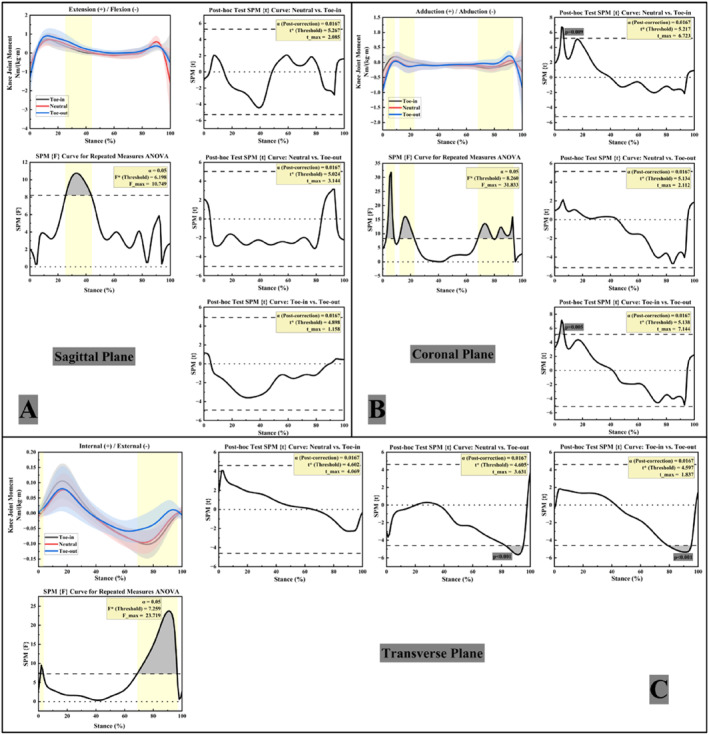
Right knee joint moments in three planes. (A) Knee joint sagittal plane moments; (B) knee joint coronal plane moments; (C) knee joint transverse plane moments.

Figure 5 shows the variations in knee joint moments of FFF participants during walking under the three FPA conditions. In the sagittal plane (Figure 5A), knee flexion–extension moments showed a significant main effect of FPA during 26%–44% of stance; however, post hoc paired t‐tests revealed no significant pairwise differences (*p* < 0.05 for the main effect).

In the coronal plane (Figure 5B), knee adduction–abduction moments showed a significant main effect of FPA during 2%–9%, 12%–23%, and 69%–94% of stance. Post hoc paired t‐tests indicated that the toe‐in gait exhibited significantly greater KAMs than the neutral condition during 6%–7% of stance and greater than the toe‐out condition during 3%–7% (*p* < 0.05).

In the transverse plane (Figure 5C), significant differences in knee internal/external rotation moments were observed among the three FPA conditions, with the toe‐in gait exhibiting the greatest fluctuation range in rotational moments. Knee internal–external rotation moments showed a significant main effect of FPA during 2%–4% and 69%–97% of stance. Post hoc paired t‐tests indicated that the neutral gait exhibited significantly greater external‐rotation moments than the toe‐out gait during 84%–95% of stance, and the toe‐in gait exhibited significantly greater external‐rotation moments than the toe‐out gait during 78%–95% (*p* < 0.05).

### 3.3 CNN Model Classification

In general, the CNN model was able to classify FPA gait patterns from ankle and knee kinematic–kinetic features with moderate to high accuracy across flatfoot severity levels. Classification performance was highest in the moderate flatfoot group, and features from the ankle coronal plane and knee transverse plane contributed most to discriminative power.

**FIGURE 6:**
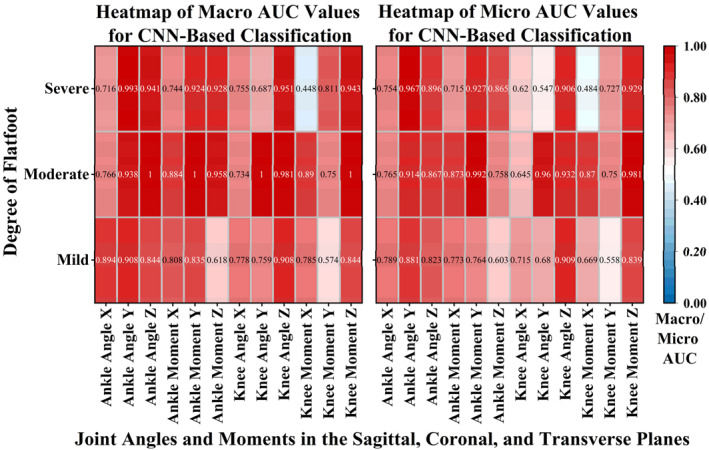
Macro‐AUC and micro‐AUC heatmap of CNN‐based FPA classification using flatfoot severity and joint kinematics and kinetics of the ankle and knee.

The CNN model's ability to classify different FPA types is depicted in Figure 6. Macro‐AUC (macro‐averaged area under the curve) and micro‐AUC (micro‐averaged area under the curve) were used to evaluate the discriminative performance of the multiclass model. Higher AUC values (represented by deeper red color in the figure) indicate stronger classification capability for a given feature under specific gait conditions. Macro‐AUC represents the arithmetic mean of AUCs calculated for each class individually, reflecting the model's balanced performance across categories, whereas Micro‐AUC is computed based on the aggregated predictions of all samples, capturing the model's overall classification performance. Notably, certain feature‐severity combinations also showed particularly strong classification ability. For example, in the severe flatfoot group, the ankle transverse plane angle achieved a macro‐AUC of 0.941 and a micro‐AUC of 0.896, whereas the ankle coronal plane moment reached a macro‐AUC of 0.924 and a micro‐AUC of 0.927, highlighting their substantial classification advantage.

CNN performance across joints, planes, and severity groups is summarized in Table 2. Across all conditions, train macro‐F1 values obtained from the single train–test split ranged from 0.17 to 0.95 and test macro‐F1 values ranged from 0.07 to 0.92. The ΔF1 (train–test) ranged from −0.10 to 0.30. In the 5‐fold cross‐validation evaluation, CV train F1 (mean) values ranged from 0.25 to 0.93 and CV validation F1 (mean) values ranged from 0.19 to 0.66. The ΔF1 (CV mean) was between −0.09 and 0.12 across all conditions. Similar patterns across severity levels and biomechanical planes were observed in both the single train–test split and the 5‐fold cross‐validation results. Across all conditions, train macro‐F1 values obtained from the single train–test split ranged from 0.17 to 0.95, and test macro‐F1 values ranged from 0.07 to 0.92. The ΔF1 (train–test) ranged from −0.10 to 0.30. In the 5‐fold cross‐validation evaluation, CV train F1 (mean) values ranged from 0.25 to 0.93 and CV validation F1 (mean) values ranged from 0.19 to 0.66. The ΔF1 (CV mean) was between −0.09 and 0.12 across all conditions. Similar patterns across severity levels and biomechanical planes were observed in both the single train–test split and the 5‐fold cross‐validation results. The results and data regarding the classification performance of FPA bait pattern based on CNN model across different flatfoot severities can be found in Supporting Information S1: Table S2. Supporting Information S1: Table S3 reports the complete fold‐level and mean 5‐fold CV metrics, including train/validation macro‐F1 and ΔF1, for each joint–plane–severity condition.

**TABLE 2:** Performance of the CNN for multiclass FPA classification across joints, planes, and AI severity groups, including single train–test and 5‐fold cross‐validation metrics.

Joint plane	Severity	Train F1	Test F1	ΔF1 (Train–test)	CV train F1 (mean)	CV Val F1 (mean)	ΔF1 (CV mean)
Ankle angle sagittal plane	Mild	0.44	0.38	0.06	0.29	0.22	0.07
	Moderate	0.60	0.56	0.05	0.44	0.37	0.07
	Severe	0.19	0.13	0.07	0.31	0.20	0.11
Ankle angle coronal plane	Mild	0.83	0.59	0.24	0.47	0.39	0.08
	Moderate	0.82	0.60	0.22	0.31	0.36	−0.06
	Severe	0.95	0.65	0.30	0.65	0.66	−0.01
Ankle angle transverse plane	Mild	0.44	0.47	−0.03	0.47	0.39	0.08
	Moderate	0.30	0.07	0.23	0.38	0.26	0.12
	Severe	0.74	0.78	−0.04	0.47	0.39	0.07
Ankle moment sagittal plane	Mild	0.75	0.56	0.19	0.31	0.28	0.04
	Moderate	0.73	0.54	0.20	0.38	0.26	0.11
	Severe	0.87	0.57	0.30	0.34	0.30	0.04
Ankle moment coronal plane	Mild	0.67	0.48	0.19	0.27	0.26	0.01
	Moderate	0.93	0.72	0.21	0.58	0.48	0.10
	Severe	0.68	0.65	0.03	0.63	0.49	0.14
Ankle moment transverse plane	Mild	0.51	0.61	−0.10	0.26	0.24	0.02
	Moderate	0.70	0.62	0.08	0.49	0.36	0.12
	Severe	0.80	0.60	0.20	0.65	0.49	0.15
Knee angle sagittal plane	Mild	0.41	0.22	0.19	0.34	0.24	0.10
	Moderate	0.72	0.45	0.27	0.45	0.37	0.08
	Severe	0.53	0.37	0.16	0.34	0.39	−0.05
Knee angle coronal plane	Mild	0.77	0.92	−0.15	0.44	0.43	0.01
	Moderate	0.86	0.49	0.37	0.93	0.59	0.34
	Severe	0.46	0.31	0.14	0.32	0.29	0.02
Knee angle transverse plane	Mild	0.65	0.43	0.23	0.29	0.30	0.00
	Moderate	0.84	0.74	0.10	0.47	0.41	0.06
	Severe	0.93	0.77	0.16	0.81	0.62	0.18
Knee moment sagittal plane	Mild	0.36	0.19	0.17	0.35	0.36	0.00
	Moderate	0.64	0.73	−0.09	0.35	0.30	0.05
	Severe	0.68	0.63	0.05	0.35	0.25	0.11
Knee moment coronal plane	Mild	0.63	0.41	0.23	0.25	0.19	0.07
	Moderate	0.17	0.22	−0.06	0.35	0.27	0.08
	Severe	0.33	0.24	0.09	0.34	0.28	0.05
Knee moment transverse plane	Mild	0.72	0.59	0.13	0.33	0.27	0.06
	Moderate	0.94	0.64	0.30	0.51	0.45	0.06
	Severe	0.68	0.54	0.14	0.39	0.42	−0.03

## 4 Discussion

This research explored how different FPA patterns (toe‐in, neutral, and toe‐out) influence ankle and knee joint biomechanics in individuals with FFF. Additionally, a CNN model was employed to perform FFF severity classification using multidimensional joint kinematic and kinetic features under different FPA settings, aiming to explore the potential relationship between gait modulation and FFF severity. The results demonstrated that changes in FPA significantly influenced joint rotation patterns and moment generation in the lower limbs, with the most pronounced differences observed in the coronal and transverse planes. The CNN model showed good classification performance across multiple feature dimensions, suggesting that FPA modulation may play a significant role in FFF pattern recognition. The findings emphasize that FPA modulation plays a significant role in altering mechanical load patterns and motor function at the lower extremity joints in those with FFF.

### 4.1 Neutral Gait

Under the neutral gait condition, ankle joint motion remained relatively balanced across all three anatomical planes. Knee joint motion under the neutral gait condition also demonstrated moderate behavior across all three anatomical planes. In the transverse plane, the neutral gait showed the lower peak rotational moments, suggesting less mechanical load on the knee joint in terms of rotational control.

The performance of both the ankle and knee joints under the neutral gait condition highlights the advantages of a neutral FPA in maintaining overall joint stability. Its relatively balanced lowerlimb alignment and coordinated muscle recruitment strategy contribute to reduced rotational stress on the knee joint, while also minimizing inversion/eversion and pronation‐related loading at the ankle. This, in turn, helps mitigate shear and torsional forces acting on joint structures [16, 48]. Such a stable movement pattern may aid in maintaining joint control during dynamic gait and reducing the risk of injury.

### 4.2 Toe‐In Gait

Our findings indicate that under the condition of toe‐in gait, the ankle joint exhibited elevated plantarflexion angles during the early propulsion phase, which may enhance push‐off efficiency [49, 50]. In the transverse plane, significantly increased internal rotation angles were observed, accompanied by elevated internal rotation moments in both the early and mid‐to‐terminal support phases. This indicates a pronounced tendency toward pronation and heightened muscular control demands [51], which may increase muscular loading and compromise overall gait stability [52].

In our results, toe‐in walking in the sagittal plane resulted in heightened KFA and decreased knee extension moment (KEM) during early stance, a pattern consistent with Huang et al.'s findings [48]. Existing studies have suggested that enhanced knee flexion during early stance facilitates shock absorption and load transfer [53, 54]. In the coronal plane, toe‐in gait increased ankle internal rotation and early‐stance KAM, accompanied by larger rotational fluctuations at the knee, suggesting that this gait pattern may elevate medial knee compartment loading and joint pressure. This modulation in KEM may be a compensatory mechanism, a finding consistent with previous literature that has documented an inverse relationship between KAM and KFMs [55]. Previous studies have shown a strong correlation between tibial torsion and FPA [56], and toe‐in gait may induce excessive tibial internal rotation, leading to altered lowerlimb alignment and rotational imbalance [48, 57]. This imbalance could elevate shear forces in the transverse plane and undermining the stability and neuromuscular coordination of the knee joint. Therefore, this gait pattern may be unfavorable for maintaining rotational balance in individuals with FFF and could potentially increase the mechanical load on periarticular soft tissues.

### 4.3 Toe‐Out Gait

In terms of ankle joint kinematics, the toe‐out gait exhibited the smallest plantarflexion angles in the sagittal plane. Although its joint moment trends were similar to those of the neutral gait, its propulsion capability appeared relatively limited. In the coronal plane, the toe‐out gait showed the greatest range of inversion/eversion angles, with relatively lower eversion moments throughout the early and mid‐segments of stance. This suggests weaker muscular engagement in controlling coronal plane instability [58], which may increase reliance on passive stabilizing components, including ligamentous tissues and joint capsular structures [59], thereby elevating the local demand for joint stabilization. Previous studies have identified the toe‐out gait as a key contributor to excessive pronation at the subtalar joint (STJ), showing a significant positive correlation with STJ eversion [60, 61, 62]. As the STJ plays a central role in regulating coronal plane motion and maintaining dynamic stability of the medial arch, abnormal motion patterns at this joint may compromise overall foot stability [59, 63]. In the transverse plane, the toe‐out gait demonstrated the smallest internal rotation angles and the least fluctuation in rotational moments, indicating restricted rotational mobility. A study by Mousavi et al. on female runners found that a toe‐out gait pattern was associated with more pronounced calcaneal eversion, greater motion of the subtalar joint into pronation, and a larger medial longitudinal arch angle (MLAA) [62], suggesting that this gait pattern may contribute to increased arch loading and midfoot instability during both walking and running.

Under the toe‐out gait condition, the knee joint demonstrated reduced flexion angles and increased extension moments in the sagittal plane, suggesting an earlier transition into knee extension during the support phase. This finding aligns with findings from Cui et al., which noted that the toe‐out gait significantly reduced peak KFAs [64], possibly reflecting diminished flexion‐extension buffering capacity and increased loading on the knee extensors [65]. Unlike findings from studies in healthy subjects reporting a reduction in KAM [15], our results showed an increase in late‐stance the second peak of the KAM (KAM2) during toe‐out gait. This discrepancy may stem from FFF‐related arch collapse and excessive subtalar pronation [66, 67], which alter tibiofemoral alignment and shift knee stability toward active rather than passive control. As a result, toe‐out gait may redistribute rather than reduce knee loading, leading to a delayed elevation in mechanical stress in FFF individuals. However, during the knee extension phase, critical stabilizing muscles, such as the biceps femoris and sartorius, may experience reduced moment arms [68, 69, 70], limiting their capacity to maintain rotational and coronal plane stability. As a result, the anticipated reduction in KAM2 under toe‐out gait may not be realized in FFF individuals. On the contrary, delayed load transfer may actually elevate KAM2, thereby increasing localized joint pressure in the later stance phase. Additionally, Jenkyn et al. observed that in patients with medial knee OA, toe‐out gait could shift some of the KAM into knee flexion moment (KFM) by altering the lever arms of the GRF in the coronal and sagittal planes [55]. In the context of our findings, although toe‐out gait reduces the first peak of KAM (KAM1) during early stance, the elevation of KAM2 in late stance suggests that total knee loading may not be reduced but rather temporally and structurally redistributed—potentially exacerbating mechanical stress in specific regions. In the transverse plane, the knee under toe‐out gait showed a notable elevation in external rotation angle, but with relatively small fluctuations in internal/external rotation moments. The strong correlation between a toe‐out gait and external tibial rotation is a well‐documented finding in the biomechanics literature [48], along with evidence that diminished KFAs tend to correspond with enhanced tibial external rotation [48, 65, 71]. In this study, the increased tibial external rotation observed with toe‐out gait may have altered the leverage of key muscle groups involved in controlling transverse‐plane loading, thereby reducing their mechanical efficiency in generating adduction and external rotation moments. Moreover, excessive tibial external rotation may impair the functional contributions of muscles, such as the soleus muscle and the gluteal musculature, during hip extension and knee stabilization [72], further weakening the knee's active control over rotational stress.

### 4.4 CNN Model Prediction

The results indicate that changes in FPA elicit identifiable kinematic and kinetic responses at the joint level, with significant biomechanical differences observed particularly in ankle coronal plane angles, together with knee transverse plane rotational behavior. These variations reflect the strategic adaptations individuals make in gait control in response to FPA changes, highlighting the practical value of FPA as a potential intervention variable. Notably, the responsiveness to FPA adjustments differed across FFF severity levels: individuals with moderate flatfoot demonstrated the highest discriminative capacity across most feature dimensions, suggesting greater sensitivity and gait adaptability; those with severe flatfoot followed, whereas mild flatfoot showed relatively weaker responsiveness. This finding implies a possible nonlinear relationship between foot structure and dynamic gait control, offering important implications for individualized intervention planning.

In terms of model performance, both Macro‐AUC and Micro‐AUC metrics confirmed that the moderate flatfoot group exhibited the strongest overall discriminative ability across the three FPA conditions. This suggests that moderate flatfoot individuals may adopt more distinct or consistent gait adjustment strategies across FPA variations. Such sensitivity reinforces the potential for targeted FPA interventions in moderate flatfoot cases.

From the perspective of feature performance, ankle angles in the coronal plane, as well as knee angles and moments in the transverse plane, consistently yielded the highest classification accuracy across conditions. These results suggest that FPA adjustments primarily affect distal joint alignment and control, subsequently leading to proximal joint moment adaptations—reflecting a typical bottom–up kinetic chain response [73]. Notably, in individuals with severe flatfoot, ankle transverse plane angles achieved a Macro‐AUC of 0.941, whereas transverse plane knee joint moments demonstrated excellent discriminative power (F1 score ≥ 0.83) in distinguishing FPA categories. These findings further support the notion that FPA modifications substantially alter lowerlimb joint loading patterns, particularly through changes in rotational dynamics.

Moreover, specific combinations of flatfoot severity and joint degrees of freedom—for example, moderate flatfoot with knee coronal plane moments—achieved perfect classification of the toe‐out gait (F1 score = 1.0). Such highly consistent responses not only demonstrate the strong discriminative power of the CNN model but also underscore the practical relevance of FPA‐targeted gait retraining or orthotic strategies. The ΔF1 values in both the single train–test split and the cross‐validation analysis indicated mild to moderate overfitting, which is expected given the relatively small dataset and the imbalance across FPA × severity cells. The relatively small ΔF1 (CV mean) values suggest that overfitting was partly mitigated by early stopping and dropout, preventing the model from relying too heavily on training samples and promoting more stable generalization across folds. The grid‐search‐based hyperparameter tuning contributed to more stable convergence and reduced overfitting as reflected by the relatively small ΔF1 (CV mean) values. Nevertheless, classification performance in several sagittal‐plane and mild‐severity conditions was low, indicating that additional biomechanical features or larger datasets may be required to fully resolve these more subtle gait differences. Future work should include data augmentation, additional sensor modalities, and larger multicenter cohorts to further improve model robustness and generalizability.

### 4.5 Clinical Implications and Limitations

Table 3 provides an integrated summary of the key biomechanical characteristics and potential clinical implications associated with neutral, toe‐in, and toe‐out gait conditions in this cohort. When developing clinical gait interventions or prescribing orthotic strategies, practitioners should carefully consider the patient's foot structure, biomechanical response patterns, and joint control capacity. Rigid application of a uniform FPA adjustment strategy may be inappropriate. Individualized selection of suitable FPA gait patterns can help optimize lowerlimb load distribution, enhance movement stability, and potentially reduce the long‐term risk of joint degeneration and chronic injuries in the ankle and knee.

**TABLE 3:** Summary of biomechanical performance and clinical implications of different FPA gait conditions in individuals with FFF.

FPA	Outstanding performance	Clinical implications
Neutral	The most balanced joint loading patterns in this cohort.	May serve as a biomechanically favorable reference during gait modulation. Nevertheless, longitudinal intervention studies are needed to determine its clinical utility in FFF gait retraining.
Toe‐in	Enhance KFMs and ankle plantarflexion during the propulsion phase, it is also associated with increased internal rotation and inversion tendencies.	Potentially raising pronation loads and muscular compensation demands. Therefore, toe‐in gait should be applied with caution in individuals with compromised ankle‐knee stability or deficient pronation control.
Toe‐in	Reducing early‐stance KAM and increased late‐stance KAM.	May lead to inadequate STJ control and a potential risk of undesirable load transfer.

This study has several limitations. First, our cohort consisted exclusively of young adults (18–35 years) with FFF, potentially restricting the applicability of the results to broader age groups or different populations. Second, although the dominant limb was intentionally selected for biomechanical analysis to ensure consistency across FPA conditions, analyzing only one limb limits the ability to evaluate potential interlimb compensation strategies or proximal joint coordination patterns. Foot morphology symmetry analysis indicated no significant differences between the left and right feet in this cohort (Table 1); however, bilateral kinematic and kinetic responses cannot be assumed to be identical. Future studies incorporating bilateral analysis, including hip joint control, are warranted. Third, the study only examined the short‐term effects of FPA modulation on ankle and knee joint mechanics, without assessing long‐term outcomes. Moreover, the deep‐learning dataset was relatively small and the study assessed only short‐term biomechanical responses to FPA modulation; therefore, future studies with larger datasets and longitudinal follow‐up designs are needed to confirm the robustness and persistence of the observed effects. Finally, finite element analysis is widely applied in sports and clinical biomechanics [74, 75]. Future research should combine finite element modeling with high‐resolution medical imaging to further investigate how varied foot progression angles alter internal knee and ankle biomechanics in young adults with flexible flatfoot. In clinical settings, the outcomes of gait interventions could be affected by multiple individual characteristics, including foot structure, muscular capacity, and neuromotor regulation. Therefore, long‐term follow‐up studies incorporating individualized characteristics are needed to validate these results. Further inquiry should broaden the participant pool and assess the long‐term implications of individualized FPA modulation protocols.

## 5 Conclusion

This investigation provided insight into the kinematic and kinetic alterations of the knee and ankle associated with distinct FPA conditions in individuals diagnosed with FFF. Neutral gait exhibited the most balanced joint loading, suggesting potential relevance as a reference. Although the toe‐in gait showed advantages in propulsion, it also increased rotational moments in the lower limbs. Alternatively, toe‐out walking may reduce ankle stability and increase knee loading during late stance, leading to loading patterns previously linked to injury. Individuals with flat feet typically exhibit greater valgus (eversion) at the distal joints of the lower extremity. This study revealed that adopting a toe‐in gait markedly diminishes valgus alignment at both the knee and ankle, indicating that adjusting the FPA could improve joint stability. However, the effectiveness of such interventions may vary across individuals. Therefore, gait adjustment strategies should be personalized based on specific foot morphology and neuromuscular control capacity, to optimize gait performance and lower the likelihood of injury. The CNN architecture used in this work demonstrated strong performance in differentiating FPA types by analyzing joint angle and moment variations across flatfoot severity groups. It showed stable and efficient classification performance, particularly for ankle coronal plane angles and knee transverse plane variables. In addition to highlighting the biomechanical responses to FPA changes, the model accurately detected the heightened gait sensitivity in individuals with moderate flatfoot, underscoring its value in personalized movement risk assessment. These results support the feasibility of using deep learning for gait modeling and offer a strong foundation for intelligent evaluation and targeted intervention in FFF management. However, it is important to note that the present evidence reflects young adults with FFF; whether similar biomechanical responses occur in children, older adults or clinical populations remains to be confirmed in future work.

## Author Contributions

**Linxiao Shen:** conceptualization, investigation, methodology, writing – original draft, formal analysis, visualization, validation. **Dong Sun:** methodology, data curation, resources, validation, writing – review and editing, formal analysis, project administration, funding acquisition. **Yufei Fang:** data curation, resources, project administration, funding acquisition. **Zhenghui Lu:** conceptualization, investigation, methodology, visualization, validation, software, formal analysis. **Xin Li:** conceptualization, investigation, methodology, visualization, validation, formal analysis. **Yufan Xu:** methodology, visualization. **Yang Song:** conceptualization, writing – review and editing. **Chengyuan Zhu:** methodology, visualization. Xuanzhen Cen: conceptualization, writing – review and editing. **Gusztáv Fekete:** methodology, writing – review and editing. **Monèm Jemni:** methodology, writing – review and editing. **Yaodong Gu:** conceptualization, investigation, methodology, visualization, validation, software, resources, data curation, formal analysis, writing – review and editing, project administration, funding acquisition, supervision.

## Funding

This study was sponsored by the National Key R&D Program of China (Grant 2024YFC3607305), Zhejiang Province Science Fund for Distinguished Young Scholars (Grant LR22A020002), Zhejiang Provincial Key Project of Education Science Planning (Grant 2025SB084), Zhejiang Engineering Research Center for New Technologies and Applications of Helium‐Free Magnetic Resonance Imaging Open Fund Project 2024 (Grants 2024GCPY02 and 2024GCPY06), Scientific Research Fund of Zhejiang Provincial Education Department (Grant Y202559510), Ningbo Key Research and Development Program (Grant 2022Z196), Zhejiang Rehabilitation Medical Association Scientific Research Special Fund (Grant ZKKY2023001), Research Academy of Medicine Combining Sports, Ningbo (Grant 2023001), Ningbo Clinical Research Center for Orthopedics and Exercise Rehabilitation (Grant 2024L004), and K. C. Wong Magna Fund in Ningbo University.

## Ethics Statement

This study was approved by the Ethics Committee of Ningbo University (Approval No. TY2025046).

## Consent

Prior to participation, all individuals were fully briefed on the experimental protocol and provided written informed consent.

## Conflicts of Interest

The authors declare no conflicts of interest.

## Supporting information


Supporting Information S1

## Data Availability

The biomechanical data and custom code used in this study are not publicly available as they are part of an ongoing research project and may be used for future work. To protect participant privacy and preserve research integrity, individual‐level data cannot be shared at this time. However, summary data and selected analysis code may be provided by the corresponding author upon reasonable request. The data that support the findings of this study are available from the corresponding author upon reasonable request.
