# Supplementary material for: Foot Progression Angle Modulates Three‐Dimensional Lower‐Limb Biomechanics in Flexible Flatfoot: Kinematic–Kinetic Patterns and Clinical Implications
Source: J Foot Ankle Res. 2026 Jan 31;19(1):e70126. doi: 10.1002/jfa2.70126 (PMC12860459; doi:10.1002/jfa2.70126)
Supplement: Supplementary file 1 — Supporting Information S1 [file JFA2-19-e70126-s001.docx]

The initial 1D convolution operation is defined by the following equation (1):

$y\left( t \right)=\sum_{i=0}^{k-1} x\left( t+i \right)*w\left( i \right)$ (1)

Where *x(t)* is the input signal, *w(i)* represents the convolution kernel weights, *k* is the kernel size, and *y(t)* is the output feature map.

In this study, a multi-channel 1D convolution operation is applied, as defined in Equation (2):

$y(t)=\sum_{c=1}^{C} \sum_{i=0}^{k-1} x_{c}\left( t+i \right)*w_{c}(i)$ (2)

Where $C$ refers to the total number of input feature channels, $x_{c}(t+i)$represents the input value from the $c$-th channel at time step $t+i$, and $w_{c}(i)$ is the convolution kernel weight for the $c$-th channel at position $i$.

In this study, the input vector $x_{c}(t)$ consists of the following features: the degree of flatfoot $x_{1}(t)$; ankle joint angles and moments in the sagittal, coronal, and transverse planes $x_{2}$​ to $x_{7}$; and knee joint angles and moments in the sagittal, coronal, and transverse planes $x_{8}$​ to $x_{13}$. Thus, the total number of input channels is $C$=13.

The pooling layer performs downsampling to reduce the dimensionality of feature maps, thereby lowering model complexity and enhancing feature robustness. Max pooling was applied to retain the most salient features. Following the convolutional stages, the resulting feature maps were first vectorized through a flattening process. This vector was then used as input for a fully connected layer to derive the high-level feature representations necessary for classification. To enhance model generalization and curb overfitting, a dropout operation was inserted immediately after the dense (fully connected) layer. The model architecture was designed to progressively extract and compress temporal features from the input biomechanical data. It consisted of two sequential blocks, each containing a convolutional layer followed by a max-pooling layer. The features generated by these blocks were then processed by a fully connected layer. Finally, to enhance generalization and prevent overfitting, a Dropout layer was incorporated after the fully connected layer. A Dropout layer (rate = 0.5) was inserted after the fully connected layer to enhance generalization. In addition, a validation dataset was incorporated throughout the training process to track model performance and help prevent overfitting. The final classification into one of three FPA types was achieved through a Softmax function applied at the output layer.

Supplementary Table 1 presents the standardised effect sizes (Cohen’s d) for all significant SPM post-hoc comparisons, based on the corresponding significant time windows, to facilitate clinical interpretation of the magnitude and direction of the observed differences. This table is intended to support the interpretation of the results presented in Sections 3.1–3.2 of the main text.

| **Supplementary Table 1. Significant SPM time windows and corresponding window-based effect sizes (Cohen’s d) for all post-hoc comparisons** | | | | | | |
| --- | --- | --- | --- | --- | --- | --- |
| **Joint angle/**  **moment** | **Plane** | **SPM{F} significant window (% stance)** | **Comparison** | **SPM{t} significant window**  **(% stance)** | ***p-value*** | **Effect size**  **(Cohen’s d)** |
| Ankle angle | Sagittal plane | - | - | - | - | - |
|  | Coronal plane | 0–47%  70–94% | Neutral vs. Toe-in | 3–42% | *P* <0.001 | 2.30 |
|  |  |  | Neutral vs. Toe-out | - | - | - |
|  |  |  | Toe-in vs. Toe-out | 2–40%  74–91% | *P* <0.001 | -2.16  -1.40 |
|  | Transverse plane | 0-100% | Neutral vs. Toe-in | 0-100% | *P* <0.001 | -1.99 |
|  |  |  | Neutral vs. Toe-out | 5–9%  33–41%  52–100% | *P* =0.010  *P* =0.003  *P* <0.001 | 1.36  1.34  1.71 |
|  |  |  | Toe-in vs. Toe-out | 0-100% | *P* <0.001 | 3.20 |
| Knee angle | Sagittal plane | 0–18% | Neutral vs. Toe-in | - | *-* | - |
|  |  |  | Neutral vs. Toe-out | - | *-* | - |
|  |  |  | Toe-in vs. Toe-out | 4-11% | *P* =0.014 | 1.07 |
|  | Coronal plane | 0-100% | Neutral vs. Toe-in | 6-35% | *P* <0.001 | 2.72 |
|  |  |  | Neutral vs. Toe-out | - | *-* | - |
|  |  |  | Toe-in vs. Toe-out | 3-28%  56-97% | *P* <0.001 | -1.88  -2.18 |
|  | Transverse plane | 0-100% | Neutral vs. Toe-in | 0-100% | *P* <0.001 | 1.75 |
|  |  |  | Neutral vs. Toe-out | 0-75% | *P* <0.001 | -1.36 |
|  |  |  | Toe-in vs. Toe-out | 0-100% | *P* <0.001 | -1.74 |
| Ankle moment | Sagittal plane | 72–93% | Neutral vs. Toe-in | - | *-* | - |
|  |  |  | Neutral vs. Toe-out | - | *-* | - |
|  |  |  | Toe-in vs. Toe-out | 78–90% | *P* <0.001 | -1.43 |
|  | Coronal plane | 0–6% | Neutral vs. Toe-in | 0–3% | *P* =0.014 | -1.60 |
|  |  |  | Neutral vs. Toe-out | - | *-* | - |
|  |  |  | Toe-in vs. Toe-out | 0–2% | *P* =0.016 | 1.37 |
|  | Transverse plane | 4–31%  69–80% | Neutral vs. Toe-in | 6–7% | *P* =0.013 | -1.42 |
|  |  |  | Neutral vs. Toe-out | - | *-* | - |
|  |  |  | Toe-in vs. Toe-out | 10–20% | *P* <0.001 | 1.50 |
| Knee moment | Sagittal plane | 26–44% | Neutral vs. Toe-in | - | *-* | - |
|  |  |  | Neutral vs. Toe-out | - | *-* | - |
|  |  |  | Toe-in vs. Toe-out | - | *-* | - |
|  | Coronal plane | 2–9%  12–23%  69–94% | Neutral vs. Toe-in | 6–7% | *P* =0.009 | -2.01 |
|  |  |  | Neutral vs. Toe-out | - | *-* | - |
|  |  |  | Toe-in vs. Toe-out | 3–7% | *P* =0.005 | 1.97 |
|  | Transverse plane | 2–4%  69–97% | Neutral vs. Toe-in | - | *-* | - |
|  |  |  | Neutral vs. Toe-out | 84–95% | *P* <0.001 | 1.51 |
|  |  |  | Toe-in vs. Toe-out | 78–95% | *P* <0.001 | 1.47 |
| Note: Cohen’s d was computed as the mean effect size across each significant time window (preserving the sign to indicate direction of the effect). | | | | | | |

The classification performance of the CNN model, which reflect the combined performance of precision and recall in a multiclass task, are presented in Supplementary Table 2. An F1-score closer to 1 indicates better classification performance. Among the classifications based on ankle joint angle features, the coronal plane angle yielded the highest accuracy, with all three flatfoot severity groups achieving over 70% accuracy. In particular, the severe flatfoot group demonstrated the most notable performance, with F1-scores exceeding 0.73 across the board. Furthermore, the ankle's transverse plane angle was a strong classifier for severe flatfoot (Accuracy = 73.33%; F1-score ≥ 0.67). For ankle joint moment features, coronal plane data provided the highest discriminative power for moderate flatfoot (Accuracy = 87.50%; F1-score ≥ 0.67). Meanwhile, the severe flatfoot group also exhibited excellent classification results in both the coronal and transverse planes, with accuracies of 73.33% and 78.57%, respectively. Notably, in the coronal plane, the classification of the toe-out gait for severe flatfoot achieved an outstanding F1-score of 0.91. In the transverse plane, all F1-scores for severe flatfoot exceeded 0.70, indicating robust classification performance.

In the classification based on knee joint angle features, the transverse plane exhibited the best discriminative performance, with classification accuracies exceeding 75% across all flatfoot severity levels and an average F1-score of 0.67. Notably, the combination of coronal plane angles and moderate flatfoot also showed strong classification ability (accuracy = 80.00%; F1-score ≥ 0.67). This combination was particularly effective in identifying the toe-out gait, with an F1-score reaching 1.0. For knee joint moment features, the transverse plane again demonstrated superior classification performance, especially in moderate and severe flatfoot cases, with classification accuracies of 88.89% and 85.71%, respectively, and corresponding F1-scores no lower than 0.83. These results indicate the model's strong capability in recognizing gait patterns based on knee kinetics. Furthermore, certain combinations of flatfoot severity and joint degrees of freedom showed notable potential in distinguishing specific FPA types. For instance, moderate flatfoot combined with knee coronal plane moments achieved an F1-score of 1.0 in identifying the toe-out gait, while mild flatfoot combined with knee transverse plane moments yielded an F1-score of 0.93 for toe-in gait classification. These findings highlight the utility of the extracted features in distinguishing gait patterns, demonstrating their potential for gait analysis and intervention design. Additionally, A notable discrepancy between training and validation performance was observed. While the model achieved high accuracy on the training data, its ability to generalize was limited, as evidenced by the considerably lower F1 scores on the test set, indicating a degree of overfitting. To mitigate this issue, future work may consider increasing the dataset size, refining the network architecture, or applying stronger regularization techniques.

| **Supplementary Table 2.** Classification Performance of FPA Gait Pattern Based on CNN Model Across Different Flatfoot Severities | | | | |
| --- | --- | --- | --- | --- |
| **FPA** | | **Mild** | **Moderate** | **Severe** |
| **Flatfoot Severities** | | **Neutral Toe-in Toe-out** | **Neutral Toe-in Toe-out** | **Neutral Toe-in Toe-out** |
| **Ankle Angle X** | **Trial count** | 24 27 35 | 8 15 19 | 18 17 28 |
|  | **Accuracy** | 61.11% | 55.56% | 53.85% |
|  | **Precision** | 0.43 0.50 0.57 | 0.00 0.50 1.00 | 0.50 0.67 0.75 |
|  | **Recall** | 0.60 0.33 0.57 | 0.00 1.00 0.75 | 0.75 0.67 0.60 |
|  | **F1-score** | 0.60 0.25 0.78 | 0.00 0.40 0.80 | 0.44 0.00 0.67 |
|  | **Macro-F1** | 0.49 | 0.51 | 0.62 |
| **Ankle Angle Y** | **Trial count** | 28 29 30 | 10 13 13 | 21 21 28 |
|  | **Accuracy** | 72.22% | 75.00% | 78.57% |
|  | **Precision** | 0.43 0.50 0.57 | 0.00 0.50 1.00 | 0.50 0.67 0.75 |
|  | **Recall** | 0.60 0.33 0.57 | 0.00 1.00 0.75 | 0.75 0.67 0.50 |
|  | **F1-score** | 0.55 0.80 0.80 | 0.00 0.86 0.75 | 0.73 0.75 0.89 |
|  | **Macro-F1** | 0.60 | 0.72 | 0.65 |
| **Ankle Angle Z** | **Trial count** | 35 37 36 | 7 16 13 | 21 25 29 |
|  | **Accuracy** | 68.18% | 62.50% | 73.33% |
|  | **Precision** | 0.50 0.75 0.70 | 0.33 1.00 1.00 | 1.00 1.00 0.75 |
|  | **Recall** | 0.29 0.75 1.00 | 1.00 0.50 1.00 | 0.50 1.00 1.00 |
|  | **F1-score** | 0.36 0.71 0.84 | 0.00 0.67 0.86 | 0.67 0.75 0.77 |
|  | **Macro-F1** | 0.65 | 0.72 | 0.84 |
| **Ankle Moment X** | **Trial count** | 38 38 36 | 20 20 22 | 23 25 29 |
|  | **Accuracy** | 60.87% | 69.23% | 56.25% |
|  | **Precision** | 0.75 0.67 0.60 | 0.40 0.50 0.75 | 0.67 0.67 0.60 |
|  | **Recall** | 0.38 0.75 0.86 | 0.50 0.50 0.60 | 0.40 0.40 1.00 |
|  | **F1-score** | 0.67 0.67 0.46 | 0.67 0.50 0.77 | 0.36 0.55 0.80 |
|  | **Macro-F1** | 0.64 | 0.54 | 0.60 |
| **Ankle Moment Y** | **Trial count** | 36 33 35 | 9 15 13 | 22 23 29 |
|  | **Accuracy** | 57.14% | 87.50% | 73.33% |
|  | **Precision** | 0.50 0.67 0.67 | 0.50 0.67 1.00 | 0.67 0.67 0.83 |
|  | **Recall** | 0.86 0.29 0.57 | 0.50 0.67 1.00 | 1.00 0.40 0.83 |
|  | **F1-score** | 0.33 0.53 0.80 | 0.67 1.00 0.89 | 0.67 0.57 0.91 |
|  | **Macro-F1** | 0.55 | 0.72 | 0.71 |
| **Ankle Moment Z** | **Trial count** | 35 33 36 | 11 15 13 | 21 22 27 |
|  | **Accuracy** | 42.86% | 62.50% | 78.57% |
|  | **Precision** | 0.75 0.75 0.67 | 1.00 1.00 0.50 | 0.43 0.75 1.00 |
|  | **Recall** | 0.43 0.86 0.86 | 0.50 0.33 1.00 | 0.75 0.60 0.60 |
|  | **F1-score** | 0.00 0.55 0.60 | 0.57 1.00 0.57 | 0.73 0.86 0.80 |
|  | **Macro-F1** | 0.70 | 0.61 | 0.65 |
| **Knee Angle X** | **Trial count** | 35 35 33 | 20 21 21 | 18 24 24 |
|  | **Accuracy** | 47.62% | 46.15% | 35.71% |
|  | **Precision** | 0.50 0.50 0.86 | 0.40 0.75 0.25 | 0.00 0.57 0.29 |
|  | **Recall** | 0.57 0.43 0.86 | 0.50 0.60 0.25 | 0.00 0.80 0.40 |
|  | **F1-score** | 0.33 0.56 0.50 | 0.60 0.57 0.22 | 0.22 0.60 0.22 |
|  | **Macro-F1** | 0.62 | 0.45 | 0.33 |
| **Knee Angle Y** | **Trial count** | 21 18 22 | 7 6 9 | 11 12 16 |
|  | **Accuracy** | 53.85% | 80.00% | 37.50% |
|  | **Precision** | 0.67 0.80 0.80 | 1.00 0.50 1.00 | 0.33 1.00 0.25 |
|  | **Recall** | 0.50 1.00 0.80 | 0.50 1.00 1.00 | 0.50 0.33 0.33 |
|  | **F1-score** | 0.40 0.67 0.60 | 0.67 0.67 1.00 | 0.00 0.50 0.50 |
|  | **Macro-F1** | 0.75 | 0.78 | 0.40 |
| **Knee Angle Z** | **Trial count** | 37 29 36 | 13 16 15 | 17 22 29 |
|  | **Accuracy** | 76.19% | 77.78% | 78.57% |
|  | **Precision** | 0.75 0.57 0.60 | 1.00 0.75 0.75 | 0.60 1.00 1.00 |
|  | **Recall** | 0.38 0.67 0.86 | 0.33 1.00 1.00 | 1.00 1.00 0.67 |
|  | **F1-score** | 0.75 0.67 0.86 | 0.50 0.67 1.00 | 0.73 0.80 0.83 |
|  | **Macro-F1** | 0.61 | 0.74 | 0.85 |
| **Knee Moment X** | **Trial count** | 19 18 15 | 15 18 12 | 9 13 15 |
|  | **Accuracy** | 54.55% | 66.67% | 37.50% |
|  | **Precision** | 0.50 0.75 0.40 | 1.00 0.80 1.00 | 0.33 0.50 0.67 |
|  | **Recall** | 0.25 0.75 0.67 | 0.67 1.00 1.00 | 0.50 0.33 0.67 |
|  | **F1-score** | 0.44 0.80 0.50 | 0.67 0.00 0.86 | 0.00 0.00 0.67 |
|  | **Macro-F1** | 0.53 | 0.90 | 0.49 |
| **Knee Moment Y** | **Trial count** | 23 12 20 | 9 7 4 | 11 13 15 |
|  | **Accuracy** | 36.36% | 50.00% | 62.50% |
|  | **Precision** | 0.75 0.00 0.60 | 0.00 1.00 0.33 | 0.67 1.00 1.00 |
|  | **Recall** | 0.60 0.00 0.75 | 0.00 1.00 1.00 | 1.00 0.67 1.00 |
|  | **F1-score** | 0.40 0.40 0.29 | 0.00 0.50 1.00 | 0.67 0.00 0.67 |
|  | **Macro-F1** | 0.44 | 0.50 | 0.87 |
| **Knee Moment Z** | **Trial count** | 36 38 36 | 14 15 14 | 17 26 24 |
|  | **Accuracy** | 59.09% | 88.89% | 85.71% |
|  | **Precision** | 0.60 0.57 0.80 | 0.67 0.75 1.00 | 0.33 0.75 0.57 |
|  | **Recall** | 0.86 0.50 0.57 | 0.67 1.00 0.67 | 0.25 0.60 0.80 |
|  | **F1-score** | 0.53 0.93 0.20 | 0.86 1.00 0.86 | 0.83 0.89 0.86 |
|  | **Macro-F1** | 0.64 | 0.77 | 0.54 |
| Note: We quantified class imbalance by adding the number of trials per FPA × severity cell, reported class-wise Precision/Recall and Macro-F1, and provided the complete set of confusion matrices in the Supplement. | | | | |

| **Supplementary Table 3.** 5-fold cross-validation results for CNN-based FPA classification across joint planes and AI severity groups, showing fold-wise and mean Train/Validation Macro-F1 and ΔF1 values. | | | | | | |
| --- | --- | --- | --- | --- | --- | --- |
| Joint plane | Severity | Fold | Train_Macro-F1 | Val_Macro-F1 | ΔF1 (train–val) | |
| Ankle angle sagittal plane | Mild | 1 | 0.34 | 0.23 | 0.11 | |
|  |  | 2 | 0.26 | 0.16 | 0.10 | |
|  |  | 3 | 0.41 | 0.35 | 0.06 | |
|  |  | 4 | 0.27 | 0.17 | 0.11 | |
|  |  | 5 | 0.16 | 0.17 | -0.02 | |
|  |  | Mean | 0.29 | 0.22 | 0.07 | |
|  | Moderate | 1 | 0.49 | 0.35 | 0.15 | |
|  |  | 2 | 0.35 | 0.58 | -0.23 | |
|  |  | 3 | 0.51 | 0.33 | 0.17 | |
|  |  | 4 | 0.43 | 0.33 | 0.10 | |
|  |  | 5 | 0.44 | 0.26 | 0.18 | |
|  |  | Mean | 0.44 | 0.37 | 0.07 | |
|  | Severe | 1 | 0.41 | 0.14 | 0.27 | |
|  |  | 2 | 0.23 | 0.17 | 0.06 | |
|  |  | 3 | 0.31 | 0.37 | -0.06 | |
|  |  | 4 | 0.26 | 0.18 | 0.09 | |
|  |  | 5 | 0.34 | 0.17 | 0.17 | |
|  |  | Mean | 0.31 | 0.20 | 0.11 | |
| Ankle angle coronal plane | Mild | 1 | 0.40 | 0.27 | 0.13 | |
|  |  | 2 | 0.63 | 0.64 | -0.01 | |
|  |  | 3 | 0.43 | 0.34 | 0.09 | |
|  |  | 4 | 0.42 | 0.42 | 0.01 | |
|  |  | 5 | 0.46 | 0.29 | 0.17 | |
|  |  | Mean | 0.47 | 0.39 | 0.08 | |
|  | Moderate | 1 | 0.38 | 0.61 | -0.23 | |
|  |  | 2 | 0.37 | 0.33 | 0.04 | |
|  |  | 3 | 0.17 | 0.20 | -0.03 | |
|  |  | 4 | 0.42 | 0.52 | -0.10 | |
|  |  | 5 | 0.18 | 0.15 | 0.04 | |
|  |  | Mean | 0.31 | 0.36 | -0.06 | |
|  | Severe | 1 | 0.69 | 0.70 | -0.01 | |
|  |  | 2 | 0.61 | 0.65 | -0.05 | |
|  |  | 3 | 0.44 | 0.38 | 0.06 | |
|  |  | 4 | 0.75 | 0.79 | -0.05 | |
|  |  | 5 | 0.76 | 0.79 | -0.02 | |
|  |  | Mean | 0.65 | 0.66 | -0.01 | |
| Ankle angle transverse plane | Mild | 1 | 0.60 | 0.44 | 0.16 | |
|  |  | 2 | 0.59 | 0.42 | 0.17 | |
|  |  | 3 | 0.35 | 0.30 | 0.05 | |
|  |  | 4 | 0.52 | 0.42 | 0.10 | |
|  |  | 5 | 0.26 | 0.35 | -0.08 | |
|  |  | Mean | 0.47 | 0.39 | 0.08 | |
|  | Moderate | 1 | 0.41 | 0.22 | 0.19 | |
|  |  | 2 | 0.45 | 0.15 | 0.30 | |
|  |  | 3 | 0.20 | 0.24 | -0.05 | |
|  |  | 4 | 0.62 | 0.47 | 0.15 | |
|  |  | 5 | 0.21 | 0.20 | 0.01 | |
|  |  | Mean | 0.38 | 0.26 | 0.12 | |
|  | Severe | 1 | 0.27 | 0.17 | 0.11 | |
|  |  | 2 | 0.44 | 0.48 | -0.04 | |
|  |  | 3 | 0.50 | 0.45 | 0.06 | |
|  |  | 4 | 0.40 | 0.24 | 0.16 | |
|  |  | 5 | 0.71 | 0.63 | 0.07 | |
|  |  | Mean | 0.47 | 0.39 | 0.07 | |
| Ankle moment sagittal plane | Mild | 1 | 0.30 | 0.30 | 0.00 | |
|  |  | 2 | 0.34 | 0.26 | 0.07 | |
|  |  | 3 | 0.34 | 0.29 | 0.06 | |
|  |  | 4 | 0.35 | 0.35 | 0.01 | |
|  |  | 5 | 0.23 | 0.18 | 0.05 | |
|  |  | Mean | 0.31 | 0.28 | 0.04 | |
|  | Moderate | 1 | 0.45 | 0.24 | 0.21 | |
|  |  | 2 | 0.49 | 0.34 | 0.15 | |
|  |  | 3 | 0.41 | 0.25 | 0.15 | |
|  |  | 4 | 0.33 | 0.30 | 0.03 | |
|  |  | 5 | 0.20 | 0.17 | 0.04 | |
|  |  | Mean | 0.38 | 0.26 | 0.11 | |
|  | Severe | 1 | 0.43 | 0.49 | -0.05 | |
|  |  | 2 | 0.31 | 0.26 | 0.05 | |
|  |  | 3 | 0.37 | 0.29 | 0.09 | |
|  |  | 4 | 0.43 | 0.33 | 0.09 | |
|  |  | 5 | 0.16 | 0.14 | 0.02 | |
|  |  | Mean | 0.34 | 0.30 | 0.04 | |
| Ankle moment coronal plane | Mild | 1 | 0.31 | 0.18 | 0.12 | |
|  |  | 2 | 0.27 | 0.34 | -0.07 | |
|  |  | 3 | 0.24 | 0.34 | -0.10 | |
|  |  | 4 | 0.25 | 0.13 | 0.12 | |
|  |  | 5 | 0.31 | 0.32 | -0.02 | |
|  |  | Mean | 0.27 | 0.26 | 0.01 | |
|  | Moderate | 1 | 0.49 | 0.30 | 0.19 | |
|  |  | 2 | 0.64 | 0.51 | 0.13 | |
|  |  | 3 | 0.39 | 0.39 | 0.00 | |
|  |  | 4 | 0.74 | 0.55 | 0.19 | |
|  |  | 5 | 0.66 | 0.67 | -0.01 | |
|  |  | Mean | 0.58 | 0.48 | 0.10 | |
|  | Severe | 1 | 0.59 | 0.28 | 0.31 | |
|  |  | 2 | 0.67 | 0.56 | 0.11 | |
|  |  | 3 | 0.72 | 0.65 | 0.08 | |
|  |  | 4 | 0.74 | 0.66 | 0.08 | |
|  |  | 5 | 0.42 | 0.29 | 0.13 | |
|  |  | Mean | 0.63 | 0.49 | 0.14 | |
| Ankle moment transverse plane | Mild | 1 | 0.28 | 0.44 | -0.16 | |
|  |  | 2 | 0.29 | 0.17 | 0.12 | |
|  |  | 3 | 0.31 | 0.22 | 0.09 | |
|  |  | 4 | 0.26 | 0.21 | 0.06 | |
|  |  | 5 | 0.16 | 0.15 | 0.01 | |
|  |  | Mean | 0.26 | 0.24 | 0.02 | |
|  | Moderate | 1 | 0.71 | 0.59 | 0.13 | |
|  |  | 2 | 0.50 | 0.21 | 0.29 | |
|  |  | 3 | 0.50 | 0.52 | -0.02 | |
|  |  | 4 | 0.19 | 0.18 | 0.00 | |
|  |  | 5 | 0.54 | 0.33 | 0.21 | |
|  |  | Mean | 0.49 | 0.36 | 0.12 | |
|  | Severe | 1 | 0.38 | 0.16 | 0.22 | |
|  |  | 2 | 0.80 | 0.66 | 0.14 | |
|  |  | 3 | 0.84 | 0.62 | 0.22 | |
|  |  | 4 | 0.43 | 0.38 | 0.05 | |
|  |  | 5 | 0.79 | 0.65 | 0.13 | |
|  |  | Mean | 0.65 | 0.49 | 0.15 | |
| Knee angle sagittal plane | Mild | 1 | 0.47 | 0.12 | 0.34 | |
|  |  | 2 | 0.36 | 0.38 | -0.02 | |
|  |  | 3 | 0.34 | 0.17 | 0.17 | |
|  |  | 4 | 0.31 | 0.32 | -0.01 | |
|  |  | 5 | 0.23 | 0.19 | 0.04 | |
|  |  | Mean | 0.34 | 0.24 | 0.10 | |
|  | Moderate | 1 | 0.33 | 0.33 | 0.00 | |
|  |  | 2 | 0.48 | 0.19 | 0.29 | |
|  |  | 3 | 0.39 | 0.20 | 0.19 | |
|  |  | 4 | 0.51 | 0.53 | -0.03 | |
|  |  | 5 | 0.53 | 0.59 | -0.06 | |
|  |  | Mean | 0.45 | 0.37 | 0.08 | |
|  | Severe | 1 | 0.33 | 0.44 | -0.11 | |
|  |  | 2 | 0.39 | 0.23 | 0.16 | |
|  |  | 3 | 0.36 | 0.44 | -0.08 | |
|  |  | 4 | 0.34 | 0.42 | -0.08 | |
|  |  | 5 | 0.27 | 0.41 | -0.14 | |
|  |  | Mean | 0.34 | 0.39 | -0.05 | |
| Knee angle coronal plane | Mild | 1 | 0.35 | 0.45 | -0.10 | |
|  |  | 2 | 0.38 | 0.58 | -0.20 | |
|  |  | 3 | 0.32 | 0.40 | -0.09 | |
|  |  | 4 | 0.56 | 0.32 | 0.25 | |
|  |  | 5 | 0.59 | 0.41 | 0.17 | |
|  |  | Mean | 0.44 | 0.43 | 0.01 | |
|  | Moderate | 1 | 0.86 | 0.39 | 0.47 | |
|  |  | 2 | 1.00 | 0.82 | 0.18 | |
|  |  | 3 | 0.93 | 0.60 | 0.33 | |
|  |  | 4 | 0.94 | 0.56 | 0.38 | |
|  |  | 5 | 0.94 | 0.60 | 0.34 | |
|  |  | Mean | 0.93 | 0.59 | 0.34 | |
|  | Severe | 1 | 0.38 | 0.25 | 0.13 | |
|  |  | 2 | 0.35 | 0.30 | 0.04 | |
|  |  | 3 | 0.28 | 0.62 | -0.34 | |
|  |  | 4 | 0.28 | 0.10 | 0.19 | |
|  |  | 5 | 0.29 | 0.19 | 0.10 | |
|  |  | Mean | 0.32 | 0.29 | 0.02 | |
| Knee angle transverse plane | Mild | 1 | 0.33 | 0.31 | 0.03 | |
|  |  | 2 | 0.26 | 0.24 | 0.02 | |
|  |  | 3 | 0.31 | 0.24 | 0.07 | |
|  |  | 4 | 0.27 | 0.33 | -0.06 | |
|  |  | 5 | 0.29 | 0.37 | -0.07 | |
|  |  | Mean | 0.29 | 0.30 | 0.00 | |
|  | Moderate | 1 | 0.35 | 0.28 | 0.07 | |
|  |  | 2 | 0.63 | 0.48 | 0.16 | |
|  |  | 3 | 0.50 | 0.64 | -0.14 | |
|  |  | 4 | 0.49 | 0.21 | 0.28 | |
|  |  | 5 | 0.40 | 0.47 | -0.07 | |
|  |  | Mean | 0.47 | 0.41 | 0.06 | |
|  | Severe | 1 | 0.93 | 0.71 | 0.22 | |
|  |  | 2 | 0.66 | 0.71 | -0.05 | |
|  |  | 3 | 0.82 | 0.50 | 0.31 | |
|  |  | 4 | 0.82 | 0.71 | 0.11 | |
|  |  | 5 | 0.80 | 0.48 | 0.32 | |
|  |  | Mean | 0.81 | 0.62 | 0.18 | |
| Knee moment sagittal plane | Mild | 1 | 0.48 | 0.53 | -0.05 | |
|  |  | 2 | 0.26 | 0.45 | -0.18 | |
|  |  | 3 | 0.39 | 0.37 | 0.01 | |
|  |  | 4 | 0.39 | 0.24 | 0.14 | |
|  |  | 5 | 0.25 | 0.19 | 0.06 | |
|  |  | Mean | 0.35 | 0.36 | 0.00 | |
|  | Moderate | 1 | 0.61 | 0.58 | 0.03 | |
|  |  | 2 | 0.17 | 0.17 | 0.00 | |
|  |  | 3 | 0.31 | 0.24 | 0.06 | |
|  |  | 4 | 0.41 | 0.35 | 0.06 | |
|  |  | 5 | 0.27 | 0.17 | 0.10 | |
|  |  | Mean | 0.35 | 0.30 | 0.05 | |
|  | Severe | 1 | 0.20 | 0.07 | 0.12 | |
|  |  | 2 | 0.46 | 0.43 | 0.03 | |
|  |  | 3 | 0.56 | 0.20 | 0.36 | |
|  |  | 4 | 0.27 | 0.36 | -0.09 | |
|  |  | 5 | 0.28 | 0.17 | 0.12 | |
|  |  | Mean | 0.35 | 0.25 | 0.11 | |
| Knee moment coronal plane | Mild | 1 | 0.24 | 0.10 | 0.14 | |
|  |  | 2 | 0.26 | 0.18 | 0.08 | |
|  |  | 3 | 0.19 | 0.21 | -0.01 | |
|  |  | 4 | 0.28 | 0.14 | 0.14 | |
|  |  | 5 | 0.29 | 0.30 | -0.01 | |
|  |  | Mean | 0.25 | 0.19 | 0.07 | |
|  | Moderate | 1 | 0.26 | 0.40 | -0.14 | |
|  |  | 2 | 0.51 | 0.60 | -0.09 | |
|  |  | 3 | 0.24 | 0.13 | 0.11 | |
|  |  | 4 | 0.34 | 0.22 | 0.12 | |
|  |  | 5 | 0.38 | 0.00 | 0.38 | |
|  |  | Mean | 0.35 | 0.27 | 0.08 | |
|  | Severe | 1 | 0.38 | 0.30 | 0.08 | |
|  |  | 2 | 0.39 | 0.40 | 0.00 | |
|  |  | 3 | 0.26 | 0.13 | 0.13 | |
|  |  | 4 | 0.40 | 0.23 | 0.18 | |
|  |  | 5 | 0.24 | 0.36 | -0.11 | |
|  |  | Mean | 0.34 | 0.28 | 0.05 | |
| Knee moment transverse plane | Mild | 1 | 0.32 | 0.32 | 0.00 | |
|  |  | 2 | 0.33 | 0.17 | 0.16 | |
|  |  | 3 | 0.42 | 0.30 | 0.12 | |
|  |  | 4 | 0.21 | 0.28 | -0.07 | |
|  |  | 5 | 0.37 | 0.29 | 0.07 | |
|  |  | Mean | 0.33 | 0.27 | 0.06 | |
|  | Moderate | 1 | 0.77 | 0.64 | 0.13 | |
|  |  | 2 | 0.43 | 0.33 | 0.09 | |
|  |  | 3 | 0.71 | 0.74 | -0.03 | |
|  |  | 4 | 0.45 | 0.39 | 0.07 | |
|  |  | 5 | 0.17 | 0.15 | 0.03 | |
|  |  | Mean | 0.51 | 0.45 | 0.06 | |
|  | Severe | 1 | 0.28 | 0.47 | -0.19 | |
|  |  | 2 | 0.39 | 0.48 | -0.09 | |
|  |  | 3 | 0.48 | 0.32 | 0.16 | |
|  |  | 4 | 0.36 | 0.34 | 0.02 | |
|  |  | 5 | 0.45 | 0.48 | -0.03 | |
|  |  | Mean | 0.39 | 0.42 | -0.03 | |
|  | | | | | |  |
|  | | | | | |  |

| 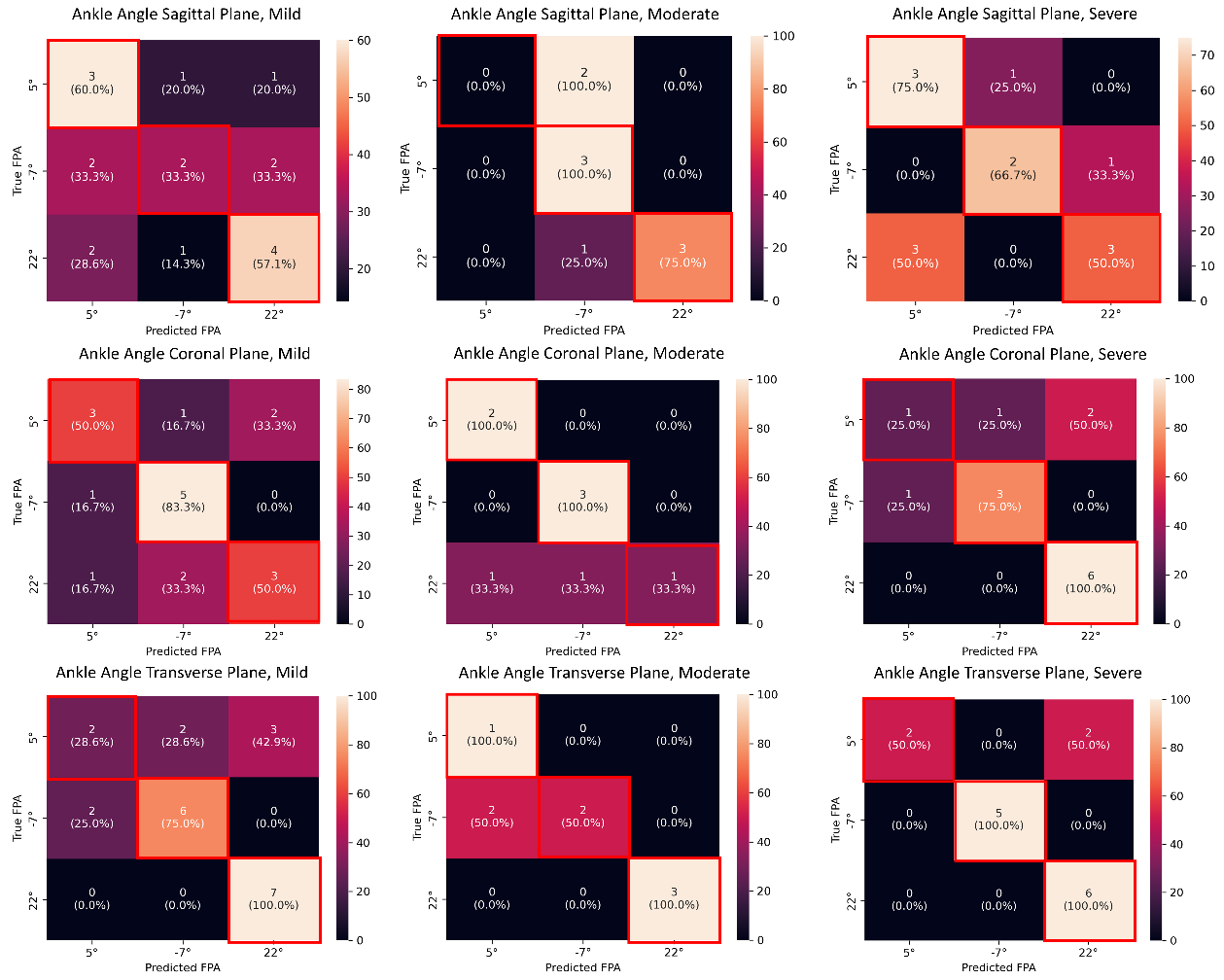 |
| --- |
| **Supplementary Figure 1.** Confusion Matrix for Ankle Angle in Each Plane |

| 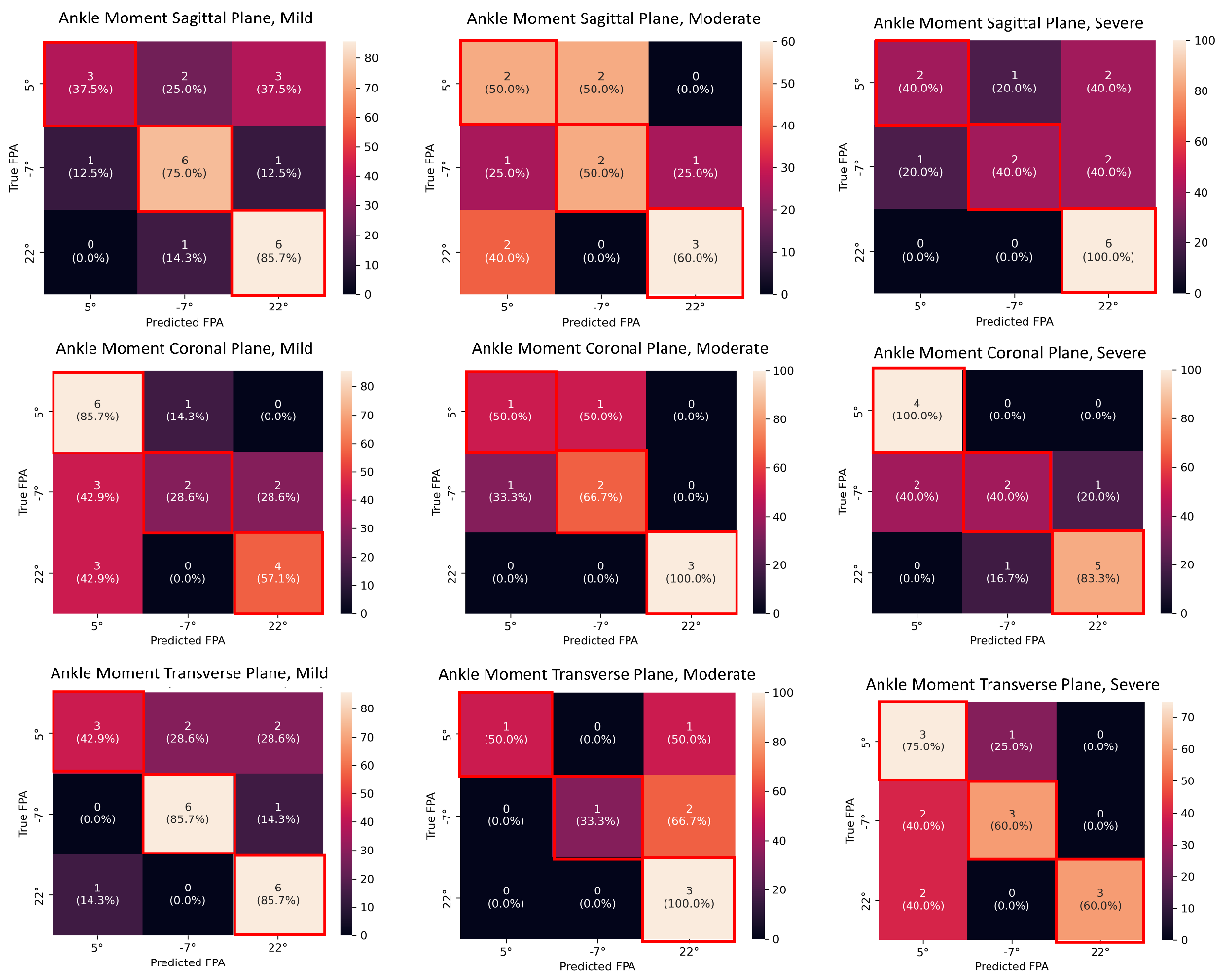 |
| --- |
| **Supplementary Figure 2.** Confusion Matrix for Ankle Moment in Each Plane |

| 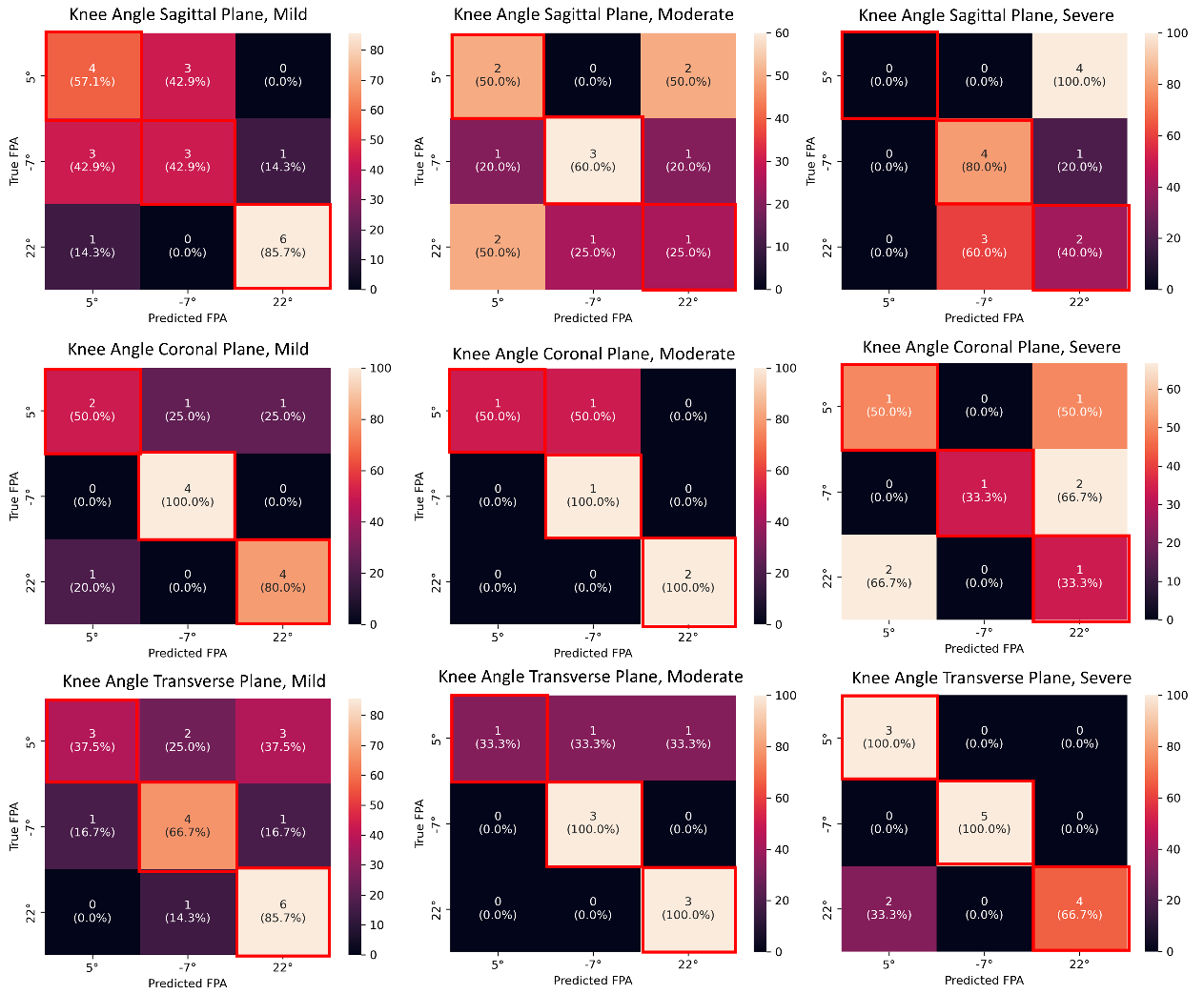 |
| --- |
| **Supplementary Figure 3.** Confusion Matrix for Knee Angle in Each Plane |

| 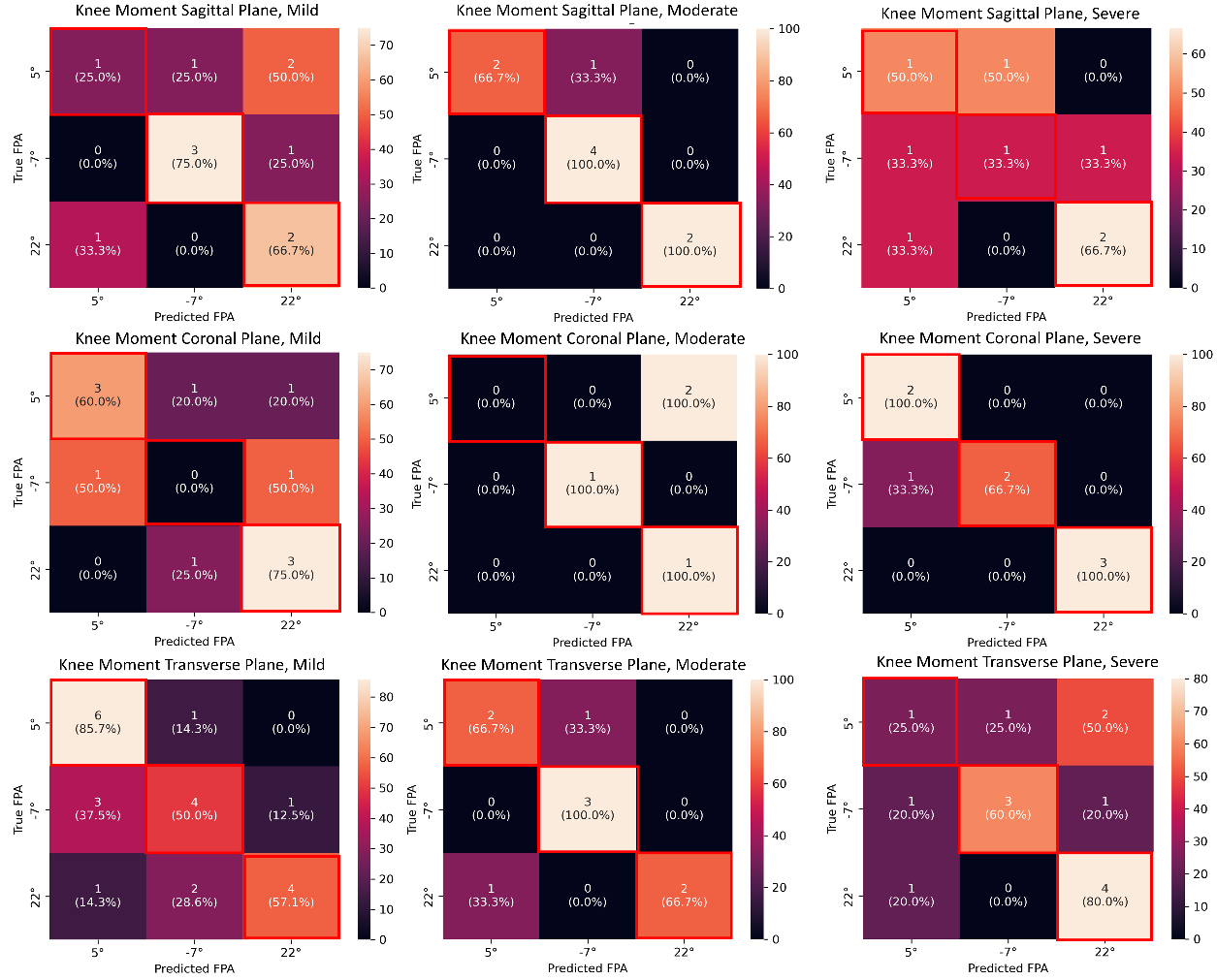 |
| --- |
| **Supplementary Figure 4.** Confusion Matrix for Knee Moment in Each Plane |

| 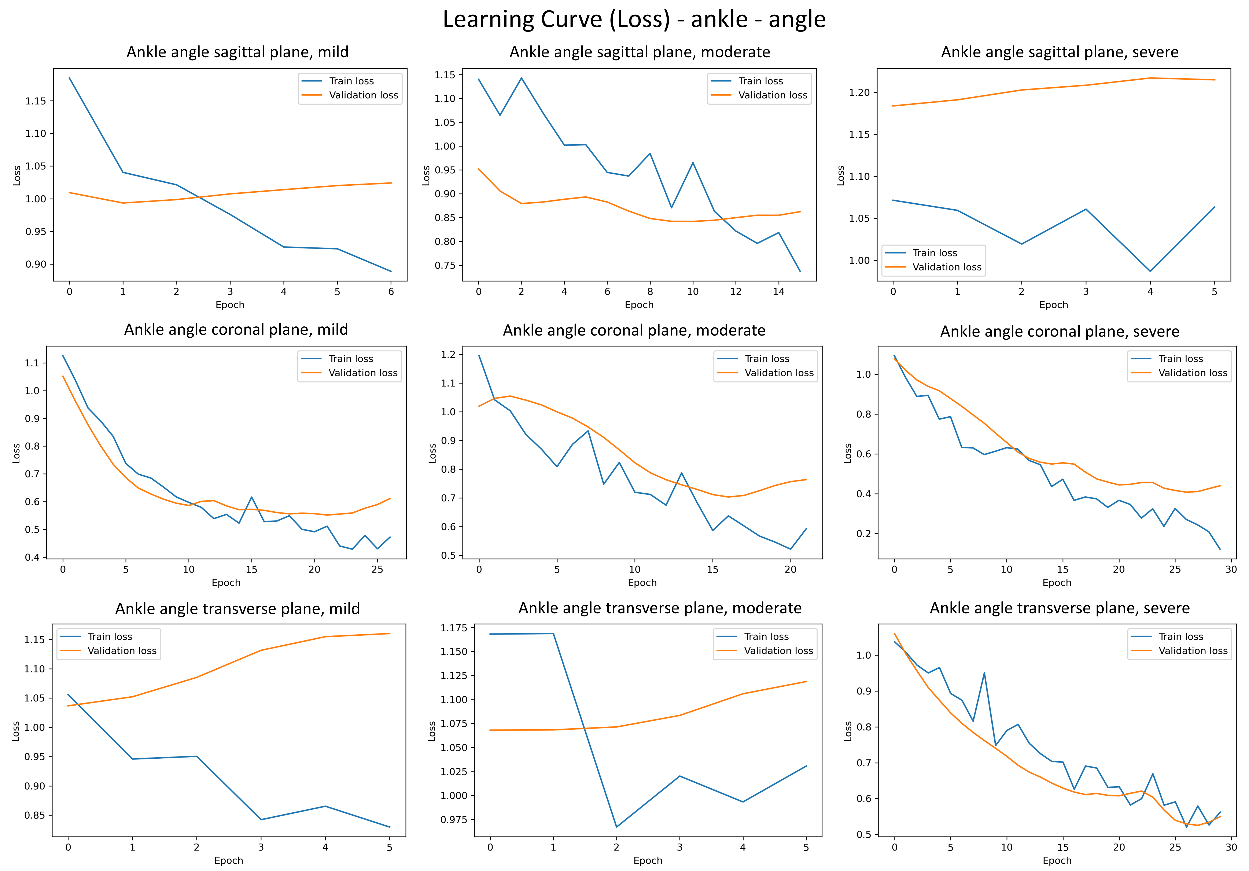 |
| --- |
| **Supplementary Figure 5.** Learning curve (loss) for ankle angle in Each Plane |

| 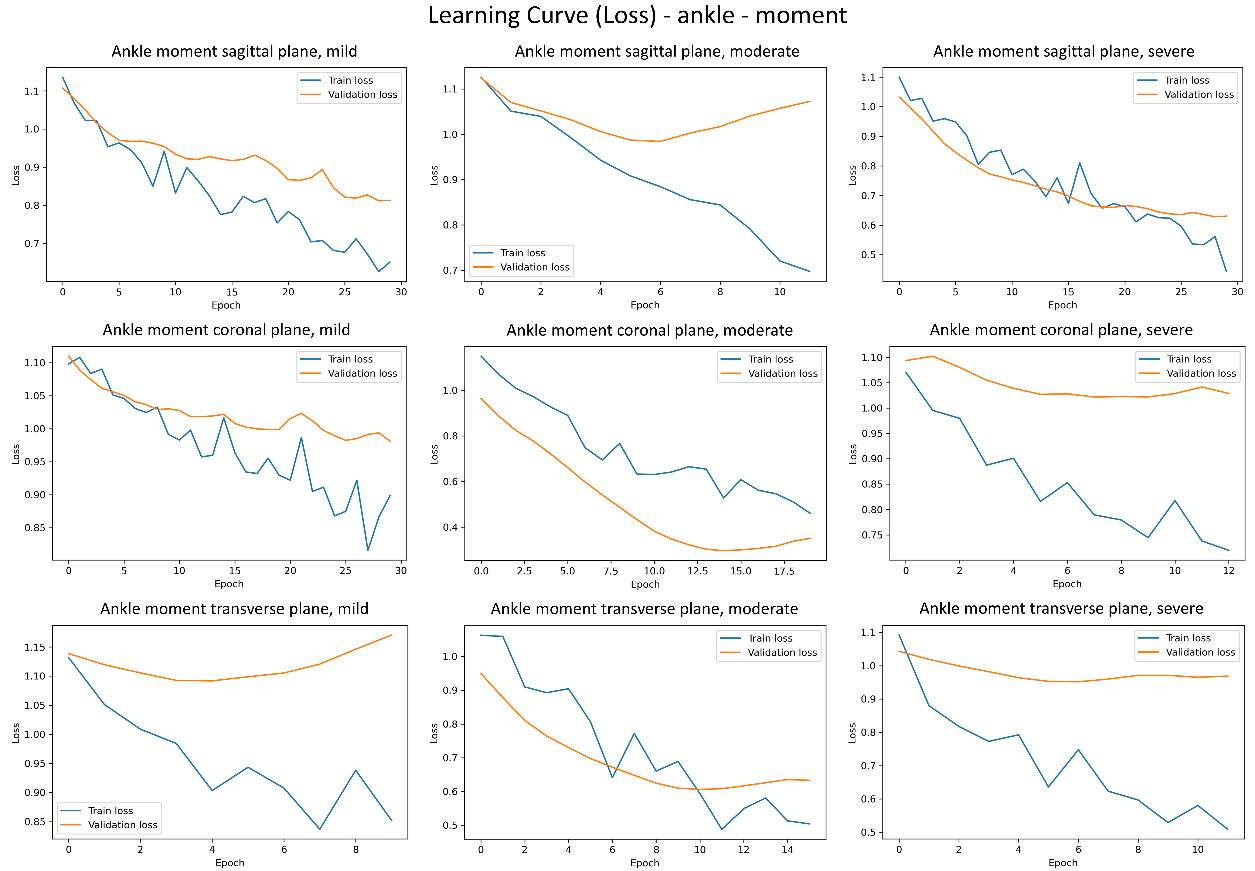 |
| --- |
| **Supplementary Figure 6.** Learning curve (loss) for ankle moment in Each Plane |

| 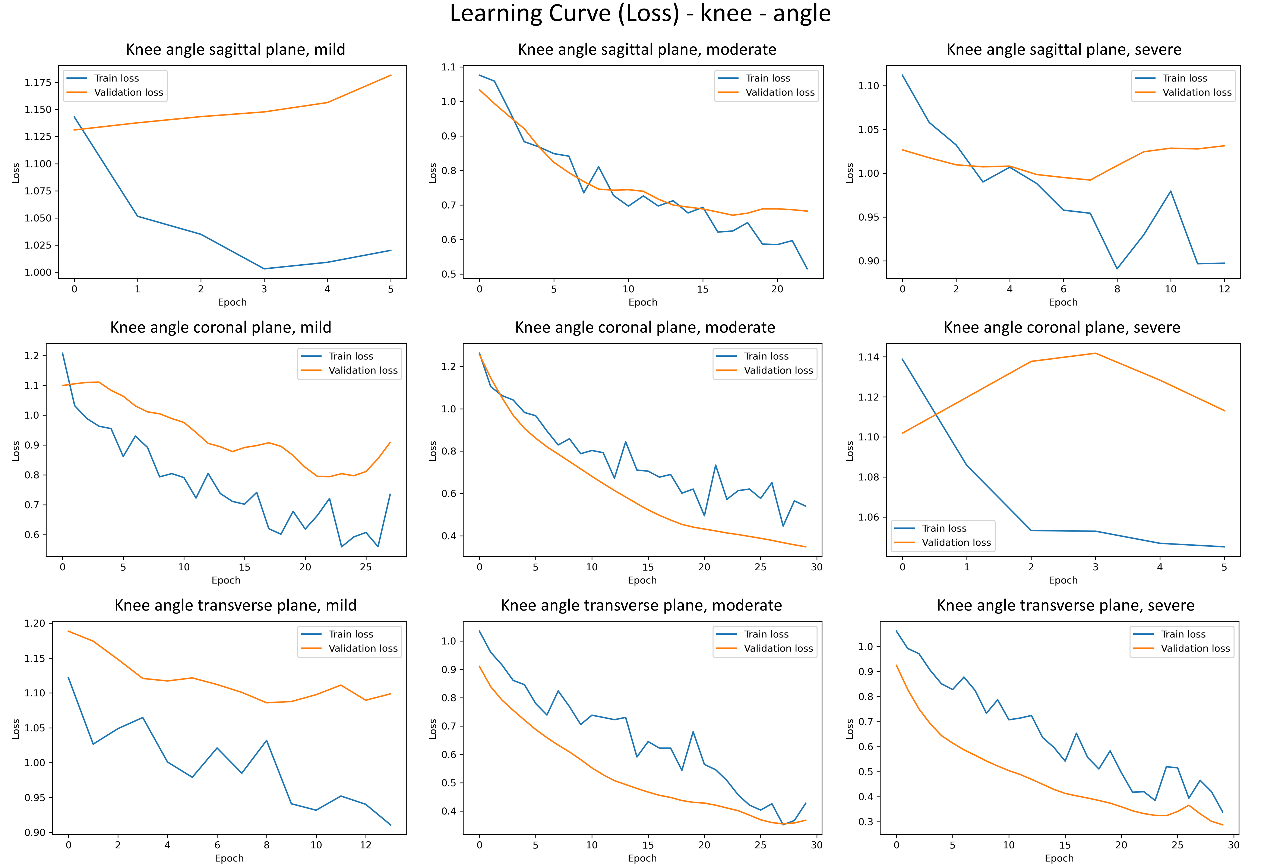 |
| --- |
| **Supplementary Figure 7.** Learning curve (loss) for knee angle in Each Plane |

| 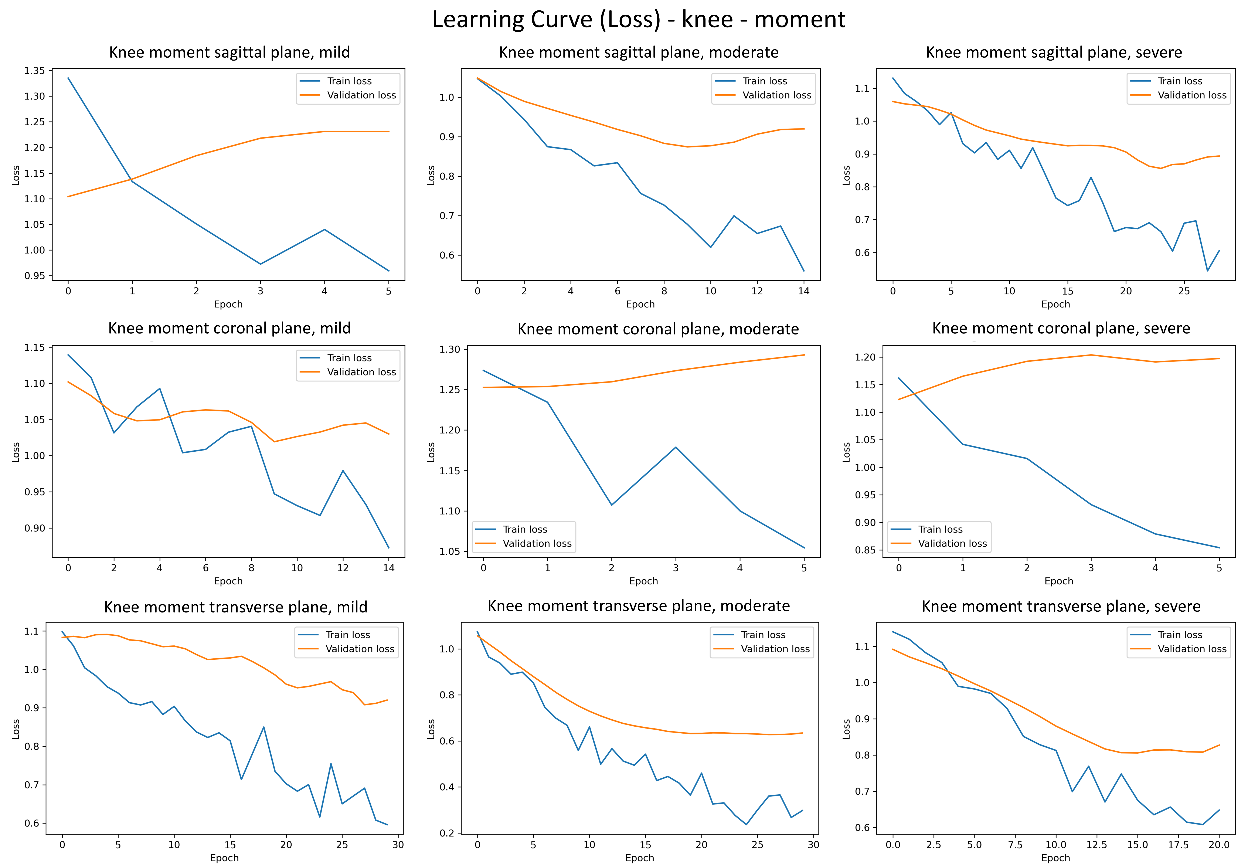 |
| --- |
| **Supplementary Figure 8.** Learning curve (loss) for knee moment in Each Plane |
